# A unified liquid chromatography-mass spectrometric approach combining targeted and untargeted analyses for community exposure profiling in wastewater

**DOI:** 10.1016/j.envint.2026.110293

**Published:** 2026-05-08

**Authors:** Pawel Lorkiewicz, Jin Chen, David Hoetker, Daymond Talley, Lauren B. Anderson, Anna Dusterhoff, Rochelle H. Holm, Ted Smith, Sanjay Srivastava

**Affiliations:** a Christina Lee Brown Envirome Institute, School of Medicine, University of Louisville, 302 E. Muhammad Ali Blvd., Louisville, KY 40202, United States; b Superfund Research Center, University of Louisville, 302 E. Muhammad Ali Blvd., Louisville, KY 40202, United States; c Division of Environmental Medicine, Department of Medicine, School of Medicine, 550 South Jackson Street, University of Louisville, Louisville, KY 40202, United States; d Center for Healthy Air Water and Soil, University of Louisville, 302 E. Muhammad Ali Blvd., Louisville, KY 40202, United States; e Louisville Metropolitan Sewer District, Morris Forman Water Quality Treatment Center, 4522 Algonquin Pkwy, Louisville, KY 40211, United States

**Keywords:** Air pollution, Exposomics, Environmental exposure, Population surveillance, Sewershed, Volatile organic compounds, Wastewater-based epidemiology

## Abstract

Wastewater-based epidemiology (WBE) has strong potential for community-level environmental exposure surveillance, but current applications remain limited in chemical scope and rarely integrate biomarker discovery with targeted quantification. Here, we developed an integrated wastewater exposomics framework combining untargeted Phase II metabolite profiling with targeted measurement of established volatile organic compound (VOC) biomarkers to characterize community-level chemical exposure. Across sixteen sewersheds in Louisville Metro, Jefferson County, Kentucky, including eleven neighborhood catchments and five centralized treatment facilities, we detected 194 Phase II metabolites derived from 145 parent xenobiotics, including mercapturic acids, glucuronides, and sulfates associated with aldehydes, aromatic hydrocarbons, epoxides, and related environmental chemicals. Several metabolites were consistently detected across sites, whereas many exhibited marked spatial heterogeneity, with greater variability at the neighborhood scale. Targeted liquid chromatography-mass spectrometry analysis of VOC biomarkers similarly revealed greater variability in neighborhood catchments than in treatment-center influent. Together, these findings demonstrate that integrating untargeted and targeted approaches extends WBE beyond limited target panels, enabling broader biomarker coverage and improved spatial resolution of community-level chemical exposures. Further refinement of normalization strategies, metabolite libraries, and data integration will enhance its utility for population-level exposure assessment.

## 1. Introduction

Humans are routinely exposed to complex mixtures of chemicals originating from traffic emissions, industrial activities, tobacco smoke, consumer products, and diet. These exposures include volatile organic compounds (VOCs), polycyclic aromatic hydrocarbons (PAHs), pesticides, pharmaceuticals, phenolic compounds, and phthalates. Exposure to these chemicals has been implicated in neurological disorders, cardiopulmonary disease, hepatic dysfunction, metabolic dysregulation, immune dysregulation, and cancer (Abplanalp et al. 2017; Liao et al. 2022; McGraw et al. 2021; Riggs et al. 2022; Wahlang et al. 2019; Wan et al. 2024; Wang et al. 2024; Wang et al. 2023). Recent wastewaterbased epidemiology studies have shown that population-level exposure to pesticides, phthalates, PAHs, and related environmental pollutants can be assessed using urinary biomarkers measured in wastewater, supporting WBE as an emerging approach for environmental exposure assessment at scale (Aghaei et al. 2024; Bowes 2024; Fattore et al. 2016; Gonzalez-Marino et al. 2021; Rousis et al. 2017a; Rousis et al. 2017b; Styszko et al. 2025). Characterizing the public health burden associated with chemical exposures requires biomonitoring approaches capable of capturing the diversity and dynamics of real-world chemical mixtures (Bowes 2024). However, existing monitoring systems provide an incomplete picture of exposure. Most programs focus on a limited set of predefined target analytes, leaving many exposure-relevant chemical classes undercharacterized or entirely absent from routine surveillance, thereby limiting the ability to capture the full spectrum of population exposures (Gasparetto et al. 2025). Although individual-level biomonitoring using blood or urine offers detailed exposure assessment, it is costly, invasive, and typically restricted to relatively small cohorts. National surveillance efforts, such as the National Health and Nutrition Examination Survey (NHANES), provide valuable population-level trends but lack the spatial and temporal resolution needed to assess community-specific exposures or shortterm variability (National Center for Health, 2025). Consequently, these approaches are insufficient to detect neighborhood-level heterogeneity in exposure within urban environments.

At the community level, publicly available Toxic Release Inventory (TRI) data report releases of selected organic chemicals including 1,3-butadiene, acrylonitrile, N,N-dimethylformamide, and benzene. However, these data are not available in near real time, do not reflect internal exposure burden, and may miss diffuse or unreported sources. These limitations underscore the need for scalable approaches with improved temporal and spatial resolution for population-level exposure assessment.

Following inhalation, ingestion, or dermal absorption, environmental chemicals undergo biotransformation through Phase I and Phase II metabolic pathways (Chen et al. 2025; Croom 2012; Testa 2007; Xie et al. 2023). Phase I reactions modify parent compounds through oxidation, reduction, or hydrolysis. Phase II conjugation reactions generate water-soluble metabolites, primarily glucuronides (GAs), sulfates (SAs), and mercapturic acids (MAs), that are readily excreted in urine. These conjugated metabolites serve as specific and integrative biomarkers of internal exposure (Alwis et al. 2012; Chen et al. 2025; Srivastava et al. 2025). Because community wastewater contains pooled metabolic signatures from large populations, it provides a unique opportunity to assess population-level exposures through sewage analysis (Scherer et al. 2025). However, most wastewater-based epidemiology (WBE) studies have focused on parent compounds or a limited number of known biomarkers, rather than broadly characterizing Phase II biotransformation products. As a result, the feasibility of measuring diverse classes of conjugated metabolites at the community scale remains poorly established.

Wastewater-based epidemiology (WBE) provides an analytical framework for considering these pooled biomarkers as anonymous indicators of community exposure. Initially developed to monitor drug consumption and infectious disease dynamics, WBE has demonstrated scalability and public health utility across diverse applications (Bowes 2024; Fattore et al. 2016; Holm et al. 2022; Kilaru et al. 2023; Kumar et al. 2022; Meyer et al. 2024; Robins et al. 2022; Scherer et al. 2025; Singer et al. 2023; Ting et al. 2024). More recently, proof-of-concept studies have extended WBE to environmental pollutants, detecting urinary metabolites of VOCs such as acrolein, benzene, styrene, and 1,3-butadiene, and PAH metabolites exhibiting seasonal patterns consistent with combustion sources (Kumar et al. 2022). These findings emphasize the potential utility of WBE for identifying and assessing community environmental health risks.

Although these studies demonstrated the feasibility of applying WBE to environmental pollutants, they remained limited in scale, chemical breadth, and integration of untargeted and targeted analytical approaches. They also did not establish whether wastewater could be used to resolve exposure differences across neighborhood-scale catchments or capture the integrated exposure burden of communities. In particular, applications of WBE to VOC- and phenolic compound-derived metabolites remain limited, leaving important classes of environmentally relevant exposure biomarkers insufficiently characterized in wastewater. Untargeted xenobiotic metabolite profiling is uncommon and rarely integrated with targeted quantitative assays. Consequently, critical information on spatial heterogeneity, exposure mixtures, and communitylevel disparities remains insufficiently characterized. Together, these limitations indicate the need for scalable frameworks for wastewater chemical surveillance and provide a robust framework for populationlevel exposure assessment. To address these gaps, we established an integrated wastewater exposomics framework combining untargeted and targeted analytical approaches. We profiled 194 Phase II metabolites derived from 145 parent xenobiotics, including mercapturic acids, glucuronides, and sulfates, across sixteen demographically diverse neighborhood scale sewershed catchments in the Louisville Metro- Jefferson County area, Kentucky, USA. Detection frequency analyses distinguished metabolites consistently present across most locations (≥75%) from those detected sporadically (≤25%). In parallel, a targeted liquid chromatography-tandem mass spectrometry (LC-MS/MS) assay quantified 29 established VOC biomarkers, including Phase I metabolites not captured by untargeted profiling. Together, these complementary approaches substantially expand the scope of wastewater chemical surveillance and provide a robust framework for population-level exposure assessment.

## 2. Materials and methods

### Study Area and Sewershed Description:

**Fig. 1.**
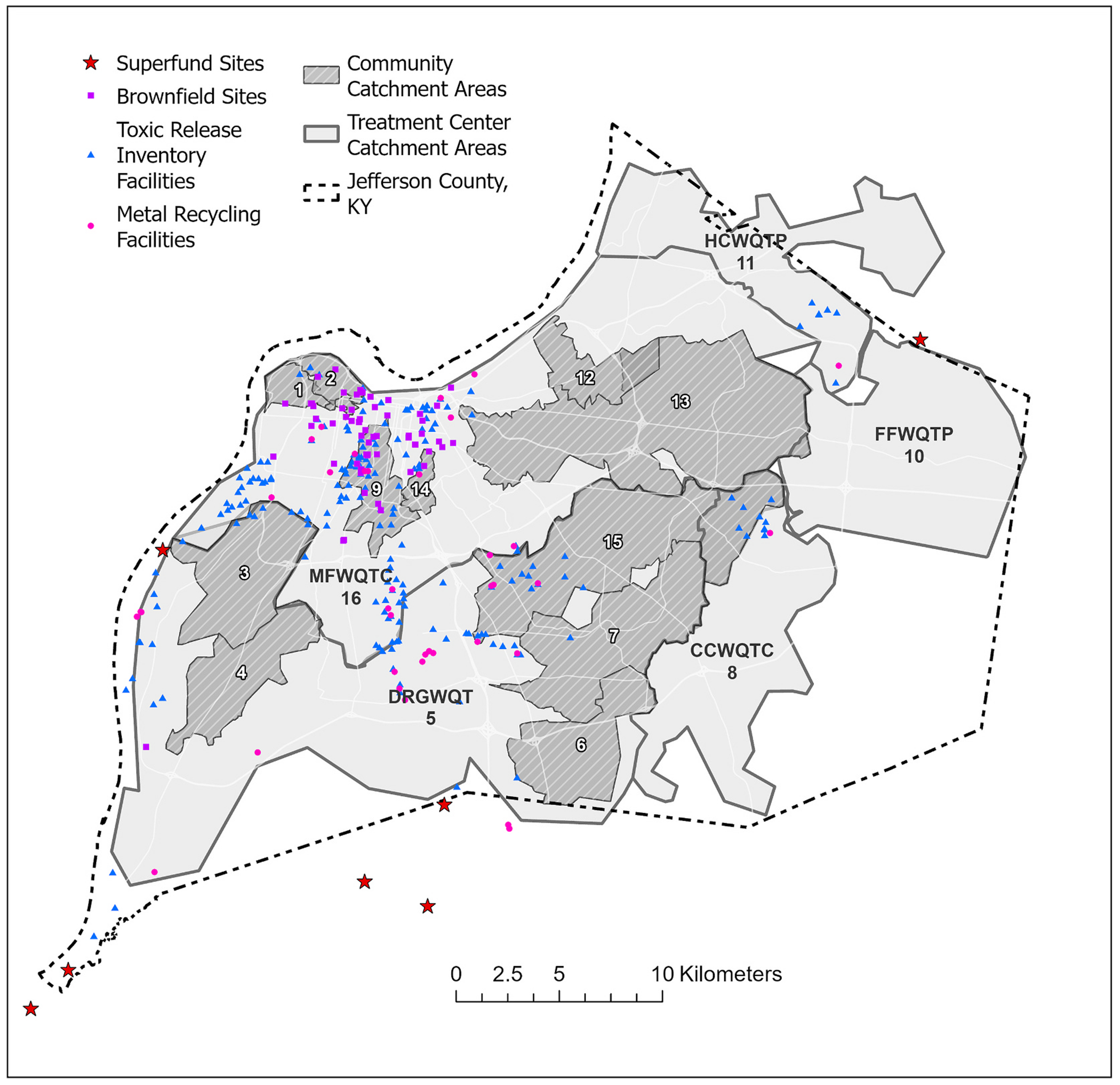
Location of wastewater sampling sites in Louisville Metro-Jefferson County, Kentucky, USA. The study included 5 wastewater treatment centers and 11 neighborhood catchments. The mapped area also includes Superfund sites, Brownfield sites, Toxic Release Inventory facilities, and metal recycling facilities that may contribute to air pollution.

The study was conducted in Louisville Metro-Jefferson County, Kentucky (KY), USA, a combined city-county region with a history of air pollution concerns (Fig. 1; Table S1). The sampled sites represent a diverse population in terms of income, race, ethnicity, and population size, with detailed demographic characteristics for each study location provided in Table S1. Site selection also emphasized representation of minority and low socioeconomic status communities (Yeager et al., 2021). Upstream, neighborhoodlevel, influent catchments accounted for 11 sites, situated within two of the county’s largest water quality treatment centers (WQTCs) (Holm et al., 2022) while the remaining five sites reflect WQTC influent. No episodic hazardous material exposures were observed in Jefferson county during the study period or the 4 weeks preceding sample collection.

### Wastewater Sample Collection Procedures:

Wastewater samples (N = 64; 16 sites × 4 weeks) were collected weekly between February 21 and March 14, 2022, with each site contributing a 24-hour time-weighted composite sample. At sample collection, a 125 mL aliquot from the composite sampler was transferred into polyethylene terephthalate bottles, stored on ice during sampling and transportation, and delivered to the University of Louisville as described previously (Holm et al., 2022; Yeager et al., 2021). The samples were stored at −80°C until analysis.

### Chemicals and Reagents:

Analyte standards and their stable isotopelabeled analogs (internal standards, IS; Table S2) were procured from LGC Standards (Manchester, NH) and Sigma-Aldrich, Inc. (St. Louis, MO). Ammonium acetate and ammonium hydroxide were obtained from Sigma-Aldrich, Inc. (St. Louis, MO). UHPLC-MS grade acetonitrile, water, and methanol were purchased from Thermo Fisher Scientific Inc. (Waltham, MA).

### LC-MS analytical platforms and workflows for wastewater analysis.

#### Sample Preparation & Solid-Phase Extraction:

Wastewater samples were processed after solid-phase extraction (SPE). First, 18 mL of each wastewater sample was mixed with 2 mL ammonium acetate (120 mM), spiked with 0.02 mL IS solution, and filtered through a 0.22 μm cellulose acetate filter (2000 g for 15 min; Amicon, Millipore-Sigma, St. Louis, MO). For SPE, Oasis MAX 20 cc Vac RC cartridges (60 mg Sorbent per Cartridge, 30 μm particle size; Waters, Milford, MA) were conditioned with 1 mL methanol, followed by 1 mL 18.2 MΩ•cm water. The filtered samples were vacuum pulled through the polymeric mixed-mode sorbent. Weakly bound compounds were removed by washing with 1 mL of 5% ammonium hydroxide in water, followed by 1 mL methanol. Analytes were then eluted into LC-MS sample tubes using 1 mL of 5% formic acid in methanol. To prevent complete solvent evaporation and potential analyte loss, 0.05 mL of water was added before evaporating the eluate under a nitrogen gas (N_2_) manifold (Thermo Fisher). The samples were then reconstituted with 0.2 mL of 0.1% formic acid and stored at −80°C until analysis.

#### Untargeted Phase II Metabolite Profiling by LC-QTOF-MS:

Untargeted profiling of Phase II metabolites in wastewater was performed using a Waters Acquity I-Class UPLC coupled to a Waters Synapt XS HDMS operated in electrospray negative ion mode as previously described (Chen et al. 2025). Samples were injected onto a Waters Acquity Premier HSS T3 VanGuard FIT column (2.1 × 100 mm, 1.8 μm) maintained at 45°C with a flow rate of 0.55 mL/min. The mobile phases consisted of solvent A (0.1% formic acid in water) and solvent B (0.1% formic acid in acetonitrile). The gradient program started at 0% B, increased to 23% B over 7.30 min, ramped to 95% B over 2.40 min, held at 95% B for 1.60 min, returned to 0% B over 0.05 min, and was reequilibrated for 1.95 min, giving a total run time of 13.30 min. The capillary voltage was set to 1.0 kV, source temperature to 112°C, desolvation gas flow to 1100 L/h at 650°C, and cone gas flow to 125 L/h. Sodium formate was used for external calibration prior to each run, and leucine enkephalin (m/z 554.2620) was infused as the lock mass during acquisition. Data were acquired in elevated-energy mass spectrometry (MS^E^) mode over an m/z range of 50–700 Da with a 0.15 sec continuum scan time. Alternating low- and high-energy scans were collected, with collision energy off in the low-energy channel and a ramp from 10 to 40 V in the high-energy channel. Raw data were processed in Waters UNIFI 1.9, applying the integrated library-guided analysis (ILGA) workflow for spectral matching against a curated spectral library of 818 Phase II metabolites (239 mercapturic acids, 431 glucuronides, 148 sulfates) from 145 parent xenobiotics, enabling detection of both known and putative biotransformation products (Chen et al. 2025). Because this approach does not require method modification, it can be readily applied for wastewater (and other biological matrices) analysis.

#### Targeted VOC Metabolite Quantification by LC-MS/MS:

VOC metabolites were measured using comprehensive VOC and tobacco alkaloid (CVT) LC-MS/MS assay as previously described (Srivastava et al., 2025). Five microliters of each sample were injected onto the column and eluted with a binary solvent system, where Solvent A consisted of 15 mM ammonium acetate in water (pH 6.8) and Solvent B was 100% acetonitrile. The gradient elution program started with 97% Solvent A and 3% Solvent B at a flow rate of 0.45 mL/min. The proportion of Solvent B was gradually increased to 5% over 1.3 min, followed by an increase to 10% over 0.7 min, then to 30% over 1.35 min, and further to 40% over 1.01 min. The gradient was then reduced to 15% B over 0.34 min, followed by a decrease to 10% B over 0.3 min, then further to 3% B over 0.36 min, where it was held for 2.64 min. Analytes were eluted from the column into the mass spectrometer and ionized in the negative ion mode under the following conditions: capillary voltage was set to 0.3 kV, source temperature was maintained at 150°C, desolvation temperature at 500°C, cone gas flow at 150 L/h, and desolvation gas flow at 1000 L/h. An automated optimization program (IntelliStart, Waters, Milford, MA) was used to determine ionization voltages, collision energies, and product ions. Multiple reaction monitoring (MRM) transitions were programmed into MassLynx 4.2 using the optimized settings. Peak integration, calibration, and quantification were performed using the TargetLynx software (Waters Inc., MA). Analytes in wastewater samples were quantified based on peak area ratio, using 10-point standard curves prepared in water. Standard curves were run before and after each batch of wastewater samples to ensure accurate quantification. In accordance with the United States Food and Drug Administration Bioanalytical Method Validation Guidance for Industry (FDA, 2018), the limit of detection (LOD) was determined as the lowest concentration at which the analyte signal was distinguishable from background noise (signal-to- noise ratio ≥ 3), while the limit of quantitation (LOQ) was defined as the lowest analyte concentration that could be quantified with acceptable accuracy and precision, and was assigned here as the lowest calibration standard with deviation from the nominal value within ± 20% (See Table S3). To evaluate precision, a pooled wastewater quality control (QC) sample was injected five times over the batch sample run, and the coefficient of variation (%CV) was calculated for each analyte (the values are listed in Table S4).

### Data Processing and Statistical Analysis:

Raw LC-QTOF-MS data were processed in UNIFI with the ILGA workflow as described (Chen et al. 2025). Following feature detection and peak picking, chromatographic signals defined by m/z and retention time were queried against an in-house library of Phase II metabolites. Annotation was performed separately for mercapturic acids, glucuronides, and sulfates, allowing detection of both known and putative conjugates. Metabolite abundances were log-transformed to stabilize variance and approximate a normal distribution. Detection frequency was calculated as the number of sampling weeks (0–4) in which a metabolite was observed at each site. Metabolites detected in all sites at least once (≥1 of 4 time points; ≥25% frequency) were considered one group, while metabolites consistently detected in all sites during most time points (≥3 of 4; ≥75% frequency) were considered a second group. Z-scores were computed from log-transformed abundances by subtracting the mean signal for each metabolite across all sites and dividing by the standard deviation.

For targeted analysis, peak areas were integrated in TargetLynx, normalized to internal standards, and quantified using external calibration curves. Concentration data were summarized using the median, interquartile range (IQR, 25th–75th percentile), and full range (minimum–maximum). Statistical differences in IQR between neighborhood catchments and wastewater quality treatment centers (WQTC) were evaluated using non-parametric tests in GraphPad Prism (version 10.4.1).

Cross-platform comparisons between untargeted and targeted data were restricted to metabolites detectable by both workflows. Untargeted responses were min–max normalized across sites to enable visual comparison with targeted concentrations. Plots were generated using GraphPad Prism. Analytical reproducibility was assessed by pooled wastewater quality control (QC) samples, which were injected five times within each batch; %CV values were calculated for all analytes to ensure precision remained within acceptable limits. For molecular network visualization, metabolite–site associations were represented as bipartite graphs. Nodes corresponded to either sampling sites or individual metabolites, with edges denoting detection events. Edge weights reflected detection frequency (0–4), and networks were color-coded by conjugation class (mercapturic acids, glucuronides, sulfates). Graphs were generated in Cytoscape (version 3.10.2), and molecular network data are provided in the Supplementary Materials.

### Evaluation of Normalization Strategies for Comparative Analyses:

To adjust for the number of individuals contributing to each community sample, we evaluated four normalization factors: cotinine, 5‑hydrox-yindoleacetic acid (5-HIAA), pepper mild mottle virus (PMMoV), and sewershed estimated population from census tract data. These factors represented complementary indicators of human contribution and sewer load. The concentrations of cotinine, 5-HIAA, and PMMoV across study sites are provided in the Supplementary Material (Table S10). Cotinine is a stable urinary metabolite of nicotine and is a well-established marker of human input (Choi et al. 2018; Rico et al. 2017). 5-HIAA is a serotonin metabolite and an endogenous indicator of total urinary excretion (Rico et al. 2017). PMMoV is a plant-derived RNA virus excreted in human feces and is a widely accepted marker of fecal strength and population connectivity (Ahmed et al. 2020). Population size was included to account for demographic differences across sewersheds (Choi et al. 2018; Ting et al. 2024; Yeager et al. 2021). The distributions of cotinine, 5-HIAA, and PMMoV across study sites are provided in the Supplementary Material. Normalization was applied to both untargeted (ILGA) and targeted (LC–MS/MS) datasets. For targeted VOC metabolite analyses, PMMoV and population size were evaluated as candidate normalization factors at WQTC sites to account for wastewater dilution within combined stormwater and sanitary sewer systems and differences in contributing population size. Flow-based normalization was not possible for neighborhood samples because flow rates were not monitored at those locations.

## 3. Results

### Profiling of Phase II Metabolites in Wastewater:

**Fig. 2.**
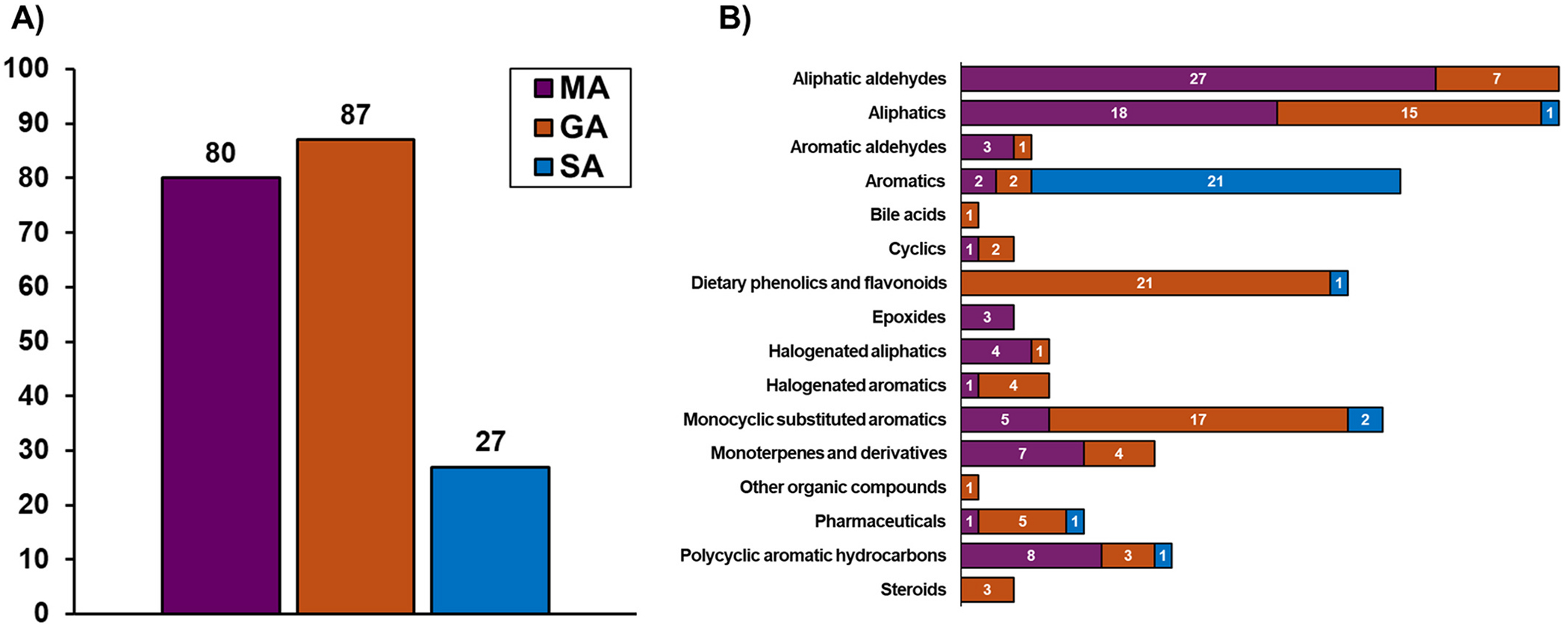
Total number of detected metabolites by conjugate class (A), including mercapturic acids (MA), glucuronides (GA), and sulfates (SA). Panel B shows the same metabolites grouped by unconjugated parent compound, with counts shown separately for each conjugate class. Analysis included 64 wastewater samples collected from sixteen sites in Jefferson County, Kentucky, including 11 neighborhood catchments and 5 wastewater treatment centers.

We analyzed 64 wastewater samples from sixteen sites in Jefferson County, Kentucky, encompassing 11 neighborhood catchments and five WQTCs. Using a previously developed integrated library-guided analysis (ILGA) LC–MS workflow (Chen et al. 2025). Phase II metabolites were profiled across all samples. In total, 194 Phase II metabolites were detected in wastewater (Fig. 2A–B), comprising 80 MAs (46 parents, 12 groups), 87 GAs (79 parents, 15 groups), and 27 SAs (20 parents, 2 groups) (Table S5). This total reflects counting repeated annotations (altogether 248 annotations *in-toto)* that differed only by retention time as a single Phase II metabolite, rather than as independent detections. (Table S6).

### Consistency and Variability of Phase II Metabolite Occurrence Across Sites:

**Fig. 3.**
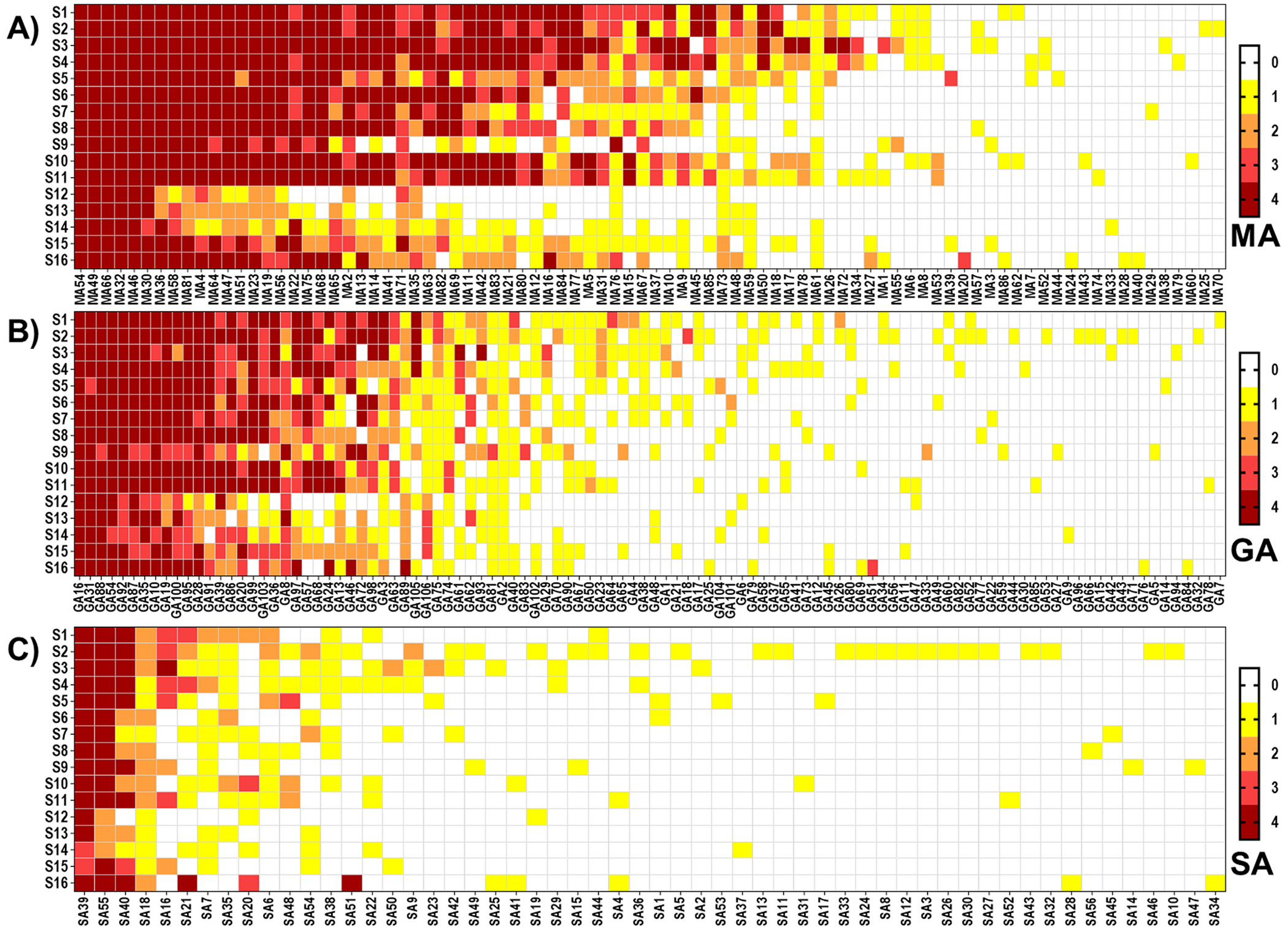
Distribution of Phase II metabolites across sixteen wastewater sites in Jefferson County, Kentucky. Heatmaps show site-level detection frequency (0–4) for (A) mercapturic acids (MA), (B) glucuronides (GA), and (C) sulfates (SA) across 64 wastewater samples, including 11 neighborhood catchments and five wastewater treatment centers.

Detection frequencies varied between sites and across time points (Fig. 3A–C). Among the three metabolite classes, GAs showed the highest degree of common detections, followed by MAs, while SAs were the least consistently observed. Sites S1, S2, S3, S4, and S11 (See Table S1 for site information) had the highest numbers of metabolites with a detection frequency > 75% (3–4) across the four sampling windows. In these locations, high-frequency detections consisted predominantly of glucuronides and mercapturic acids, with sulfates contributing only a small fraction (e.g., S1: 34 GAs, 50 MAs, 5 SAs; S3: 30 GAs, 52 MAs, 4 SAs). Lower frequency counts were recorded at S9, S12, S13, S14, and S15 (e.g., S9: 21 GAs, 23 MAs, 3 SAs). Given the sporadic nature of detected metabolites, we next identified subsets with greater spatial and temporal consistency. Specifically, 41 metabolites were observed at least once across all sites (broadly detected metabolites), and a more stringent core set of 15 metabolites was detected at each of the sixteen sites during at least three sampling windows. Despite widespread presence, several metabolites in the core group demonstrated highly localized enrichment, reaching a detection frequency of 4 in only one site; MA67 (methyl acrylate) and MA18 (4-hydroxy-2-heptenal) in S2; MA76 (2-methylpropyl carbamic acid) in S9; GA89 (4-hydroxy-2-heptenal), SA21 (tyramine), and SA51 (dopamine) in S16; and multiple compounds in S3 belonging to numerous chemical classes (e.g., MA31 (naphthalene), MA37 (acrylonitrile), GA61 (3-chloro-1,2-propanediol).

### Distinctive Metabolite Signatures Identified Across Sites:

**Fig. 4.**
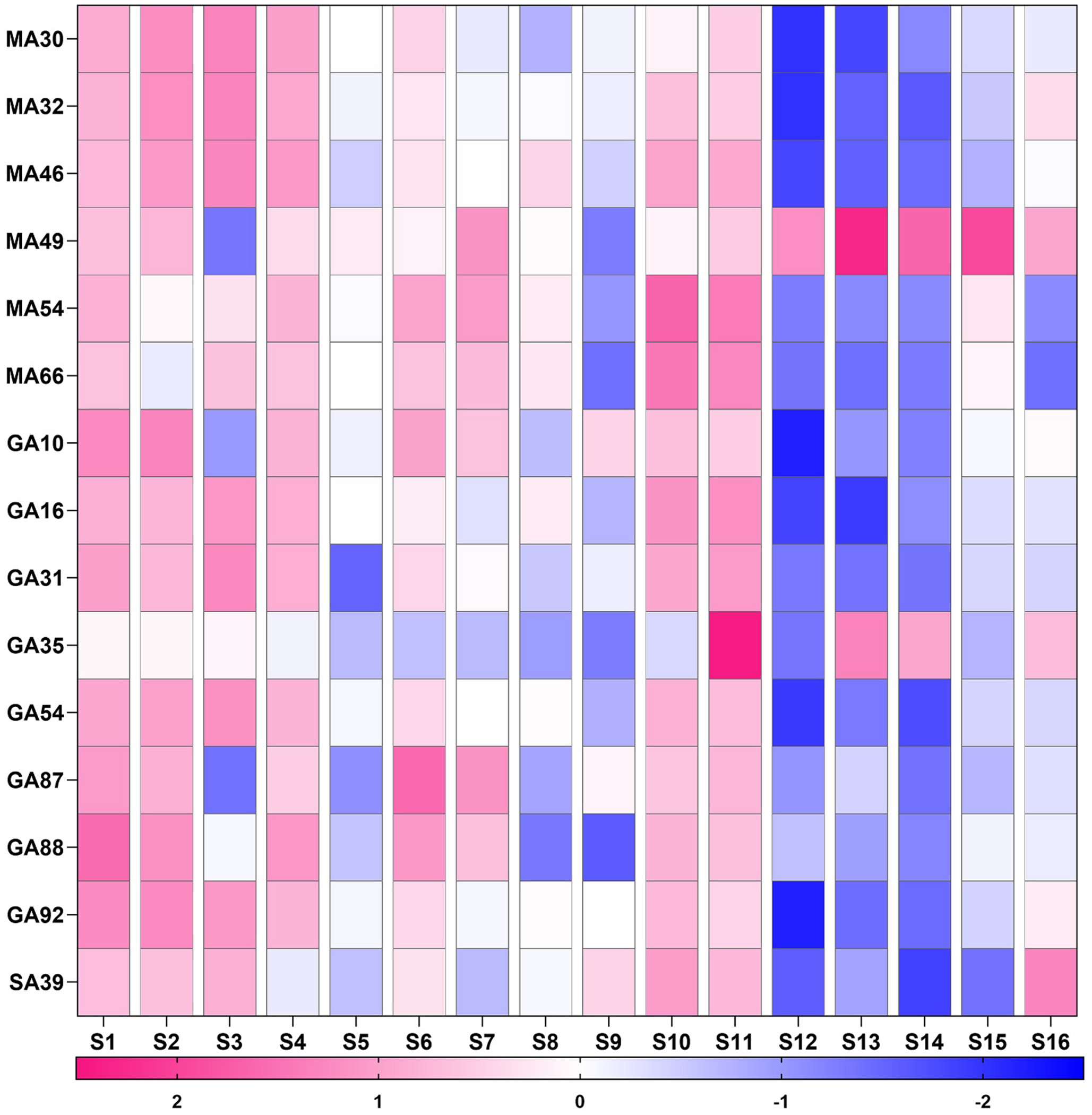
Z-score heatmap of 15 core Phase II metabolites detected at each wastewater site in at least three of four sampling periods. Columns represent sampling sites and rows represent metabolites. Z-scores were calculated from log-transformed abundances relative to the mean for each metabolite across all sites. Positive values indicate above-average abundance and negative values indicate below-average abundance.

We first examined 41 metabolites detected in each site at least once (≥1 of 4 time points; ≥25% frequency) (See Table 1). These were derived from 35 parent compounds across 14 parent groups, including 19 MAs, 16 GAs, and 6 SAs. Z-score analysis showed wide spatial variation, with sites S1, S3, and S4 showing the highest numbers of above-average compounds, while site S11 was elevated for GAs such as GA95 (4-oxo-2-heptenoic acid) and GA28 (glycerol), and S6 showed stronger signals for compounds including GA100 (dihydrocaffeic acid) and GA36 (2-ethylhexyl 3-hydroxypropanoate). Parent groups unique to this broader set, including polycyclic aromatic hydrocarbons (PAHs), monoterpenes, epoxides, and halogenated alicyclics, were among the most spatially variable. Low-abundance clusters were also evident, with sites S9, S14, and S15 and consistently showing negative z-scores. Several metabolites in this group exhibited highly localized enrichment, reaching a detection frequency of 4 in only one site, including MA48 (halogenated butene) in S1; MA67 (methyl acrylate) and MA18 (4-hydroxy-2-heptenal) in S2; MA76 (2-methylpropyl carbamic acid) in S9; GA89 (4-hydroxy-2-heptenal), SA21 (tyramine), and SA51 (dopamine) in S16; and multiple compounds in S3 belonging to numerous chemical classes (e.g., MA31 (naphthalene), MA37 (acrylonitrile), and GA61 (3-chloro-1,2-propanediol). A smaller group of 15 metabolites was consistently detected at each site during most points (≥3 of 4; ≥75% frequency) (See Table 1 and Fig. 4). Those metabolites originated from 12 unique parent compounds spanning nine parent groups: benzene/monocyclic substituted aromatics, halogenated aromatics, dietary phenolics and flavonoids, aliphatic aldehydes, pharmaceuticals, halogenated aliphatics, other aliphatics, aromatic aldehydes, and other aromatics. This subset included six MAs, eight GAs, and one SA.

Several parent compounds contributed more than one metabolite to this group, including vanillin (GA87, GA88), 1,3-butadiene (MA46, MA54), and acetaminophen (MA30, A39). Z-score heatmaps for this subset shown in Fig. 4 revealed distinct spatial trends, with sites S1, S2, S3, and showing the highest positive z-scores across several GAs and MAs, and S9 and S15 showing broadly negative values. The only SA in this set (SA39 (acetaminophen)) was most abundant at site S4. Patterns within classes displayed heterogeneity; for example, MA46 and MA54 (1,3-butadiene) were enriched in similar subsets of sites, whereas MA30 (acetaminophen) and MA32 (N,N-dimethylformamide) showed contrasting distributions. Of the GAs, GA16 (phenylpropanoic acid), GA54 (2-methyl-5-phenylpentanol), and GA88 (vanillin) often co-occurred at high abundance in the same high-scoring sites. Partial clustering of sites was evident, with sites S1 and S3 sharing elevated values for over half the metabolites, and S9 and S15 characterized by overall low abundance. Molecular network visualizations were also used to represent the distribution of the 15 core metabolites by conjugate class (Supplementary Fig. S1). The networks highlighted strong site metabolite connectivity for S1, S2, and S3, which served as central nodes for multiple MAs and GAs, while S9 and S15 appeared peripherally with limited associations. SA39, the only sulfate in this set, was predominantly connected to site S4.

### Targeted Quantification of VOC Metabolites in Wastewater:

**Fig. 5.**
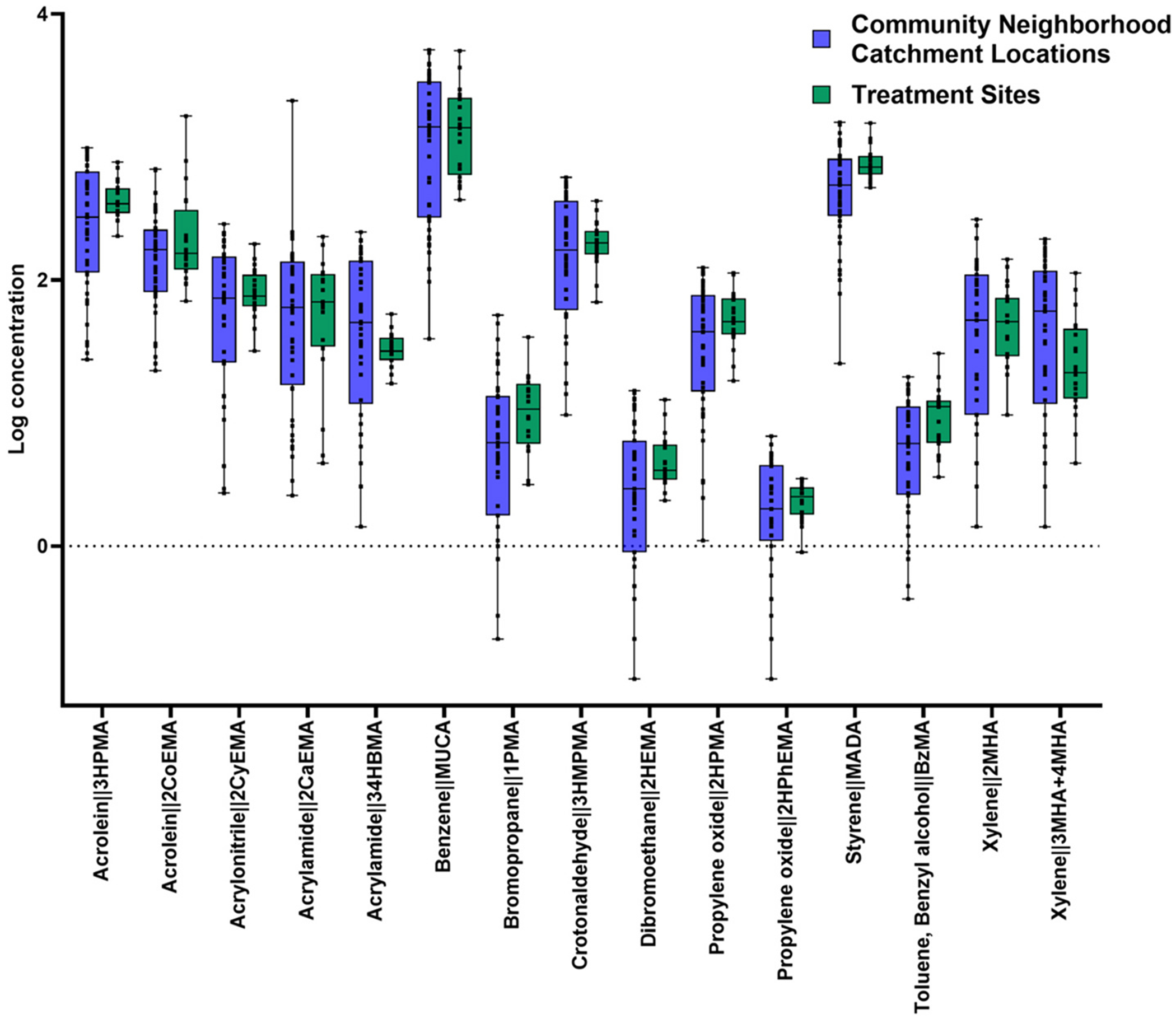
Distribution of volatile organic compound metabolite (VOCm) concentrations in wastewater samples measured by targeted analysis. Box and whisker plots represent log-transformed concentrations of selected VOCm across sixteen sampling sites, including five wastewater treatment centers and 11 neighborhood manholes. Data were acquired using the comprehensive VOC and tobacco alkaloid (CVT) liquid chromatography–tandem mass spectrometry assay.

**Fig. 6.**
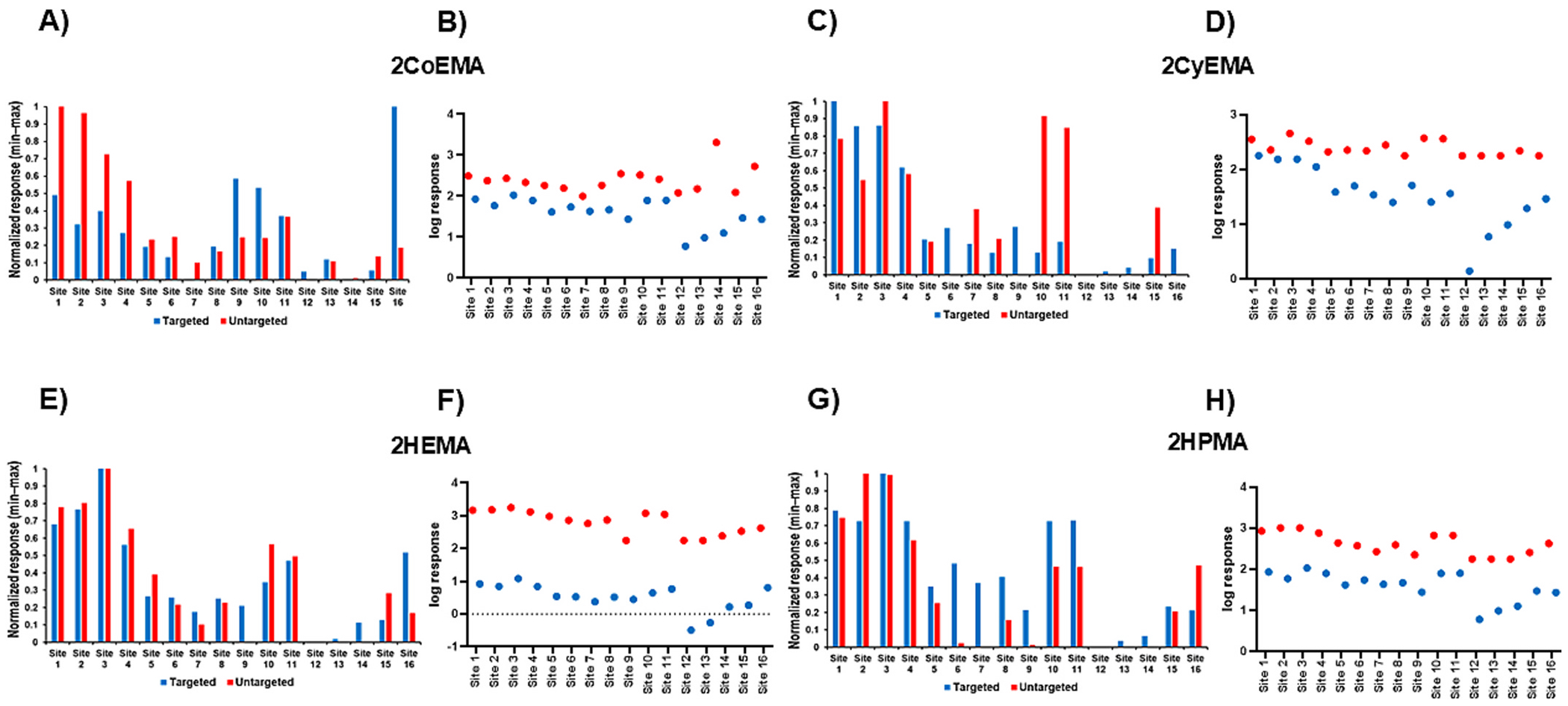
Comparison of targeted and untargeted detection for selected volatile organic compound-derived metabolites across 16 wastewater sites. Each metabolite is shown as a pair of panels, with min–max normalized responses (bar graphs) on the left and log-transformed raw responses (dot plots) on the right for targeted (blue) and untargeted (red) data. Metabolites include N-acetyl-S-(2-carboxyethyl)-L-cysteine (2CoEMA; acrolein), N-acetyl-S-(2-cyanoethyl)-L-cysteine (2CyEMA; acrylonitrile), N-acetyl-S-(2-hydroxyethyl)-L-cysteine (2HEMA; ethylene oxide), and N-acetyl-S-(2-hydroxypropyl)-L-cysteine (2HPMA; propylene oxide). Panels A, C, E, and G show normalized responses, and panels B, D, F, and H show log-transformed signal intensities. (For interpretation of the references to colour in this figure legend, the reader is referred to the web version of this article.)

We detected 15 urinary VOC metabolites prevalent at multiple sites. To more precisely quantify these and related compounds, we applied a targeted LC-MS/MS assay (Srivastava et al. 2025). This assay is designed to measure 29 urinary volatile organic compound metabolites (VOCm) (see Table S2 for full list of VOCm analyzed by the assay and Table S7 for the list of all VOCm detected and quantified in the wastewater samples), including Phase I metabolites such as MUCA (benzene) and MADA (styrene) that were not captured by untargeted profiling. The overlap between the untargeted dataset and the targeted assay is summarized in Table 2, and concentration ranges from targeted measurements are shown in Table S8. Most metabolites were detected by the targeted LC-MS/MS analysis consistently at the four time points across sites, reflecting widespread presence in wastewater. A smaller subset showed slightly lower detection frequencies (≥75%), while only a few compounds were sporadic across sites or time points. Examples include N-Acetyl-S-(2- cyanoethyl)-L-cysteine (2CyEMA; acrylonitrile), which was observed only twice at site S12, and 2-methylhippuric acid (2MHA; xylene), detected in sites S12 and S13. Among the more abundant compounds were muconic acid (MUCA; benzene), mandelic acid (MADA; styrene), and crotonaldehyde-derived 3-hydroxy-1-methylpropyl mercapturic acid (3HMPMA), followed by acrolein metabolites 3-hydroxypropyl mercapturic acid (3HPMA) and carboxyethyl mercapturic acid (2CoEMA). Full concentration ranges for detected metabolites are shown in Fig. 5. A subset of four metabolites (2CoEMA, 2CyEMA, 2HEMA, and 2HPMA) were consistently detected by both untargeted and targeted workflows and is compared directly in Fig. 6. Site-level variability for urinary VOCm was greater in neighborhood catchments than in treatment plant influents, as indicated by broader interquartile ranges and more frequent outliers (Fig. 5, Table S9). Statistically significant differences were observed for several metabolites, including acrolein (3HPMA, p = 0.006), acrylamide (2CaEMA, p = 0.005), acrylonitrile (2CyEMA, p = 0.043), crotonaldehyde (3HMPMA, p = 0.036), propylene oxide (2HPMA, p = 0.005), and toluene/benzyl alcohol (BzMA, p = 0.020). In contrast, metabolites of other VOCs exhibited relatively uniform distributions across catchments and WQTCs.

### Untargeted Profiling vs. Targeted Quantitation:

Direct comparison between untargeted profiling and targeted quantification was possible for four metabolites with sufficiently strong and consistent wastewater signals: 2CoEMA (acrolein), 2CyEMA (acrylonitrile), 2HEMA (ethylene oxide), and 2HPMA (propylene oxide) (Fig. 6). For the remaining metabolites either the concentrations were too low or the variability was too high for reliable comparison. Fig. 6 shows paired bar graphs of min–max normalized responses alongside dot plots of log-transformed intensities for 2CoEMA, 2CyEMA, 2HEMA, and 2HPMA each compound. Both approaches captured similar site-level patterns, with higher responses in sites S1–S3 and lower values in sites S9 and S15. For 2CoEMA (acrolein) and 2CyEMA (acrylonitrile), targeted and untargeted responses tracked closely across most sites, supporting concordance between workflows. 2HEMA (ethylene oxide) and 2HPMA (propylene oxide) showed greater divergence, with untargeted data yielding higher relative responses in some sites, while targeted analysis provided tighter clustering around mid-range values. Despite these differences, the two analytical approaches produced broadly similar spatial trends across the four metabolites.

### Evaluation of Normalization Strategies for Comparative Analyses:

Normalization of the untargeted dataset to any of the four factors did not reduce overall variation (see Figs. S2 and S3). Adjustments using cotinine or 5-HIAA produced only minor changes in %CVs, with a few individual sites showing localized decreases. Population-based correction increased variation by an average of about 28%, and PMMoV normalization amplified it even further, raising %CVs by more than 80% compared with unnormalized data. Together, these results show that none of the tested normalization factors improved data consistency across sites. In the targeted analyses, normalization by PMMoV and population size was also evaluated for VOC metabolites at WQTC sites (see Figs. S4–S18). Neither normalization approach produced a clear improvement in interpretability across targeted VOC metabolite profiles. Measured concentrations or abundances of cotinine, 5-HIAA, and PMMoV are provided in the Supplementary Material.

## 4. Discussion

By integrating untargeted ILGA profiling with targeted LC–MS/MS, this study supports community-scale wastewater surveillance of conjugated organic chemical metabolites. ILGA defined the occurrence and detection frequency of Phase II metabolites across sites and these results guided selection of compounds for targeted measurement and quantitation. Our framework quantifies VOC-derived metabolites rather than parent VOCs themselves to capture human internal exposure signatures while reducing potential confounding from direct industrial VOC discharges to the sewer system. Together these analyses define the range and relative stability of conjugated organic chemical metabolites detectable in wastewater. Previous quantitative and suspect-screening WBE studies have typically identified only a limited set of compounds (Foppe et al. 2021; Kumar et al. 2022; Ting et al. 2024) or focused on a narrower range of chemicals such as pharmaceuticals (Meyer et al. 2024), whereas this work achieved substantially broader coverage, identifying 194 annotated MAs, GAs, and SAs from 145 parent chemical structures. The detected metabolites represented multiple chemical groups, including aromatics, aliphatic aldehydes, phenolics, halogenated hydrocarbons, polycyclic aromatic hydrocarbons, and monoterpenes. Compounds derived from benzene-related aromatics, aldehydes, and phenolics occurred most frequently across sites. In addition to broad analytical coverage, the detection patterns observed in wastewater reveal added information about the environmental stability of conjugated metabolites. Previous studies have shown that glucuronides can undergo enzymatic hydrolysis in wastewater via β-glucuron- idases, with degradation half-lives ranging from hours to days depending on compound class and matrix conditions (Gao et al. 2018; Scherer et al. 2025; Senta et al. 2014; Shimko et al. 2022). In this study, more than one hundred distinct GAs were recovered, showing that many glucuronide conjugates remained detectable under the sampling, handling, and storage conditions used here. This agrees with prior reports in which ethyl glucuronide, morphine glucuronide, and selected steroid glucuronides were recovered from wastewater, even though some glucuronides were also shown to degrade under certain conditions (Gao et al. 2018; Scherer et al. 2025; Senta et al. 2014; Shimko et al. 2022). Together, these observations show that glucuronide recovery in wastewater depends on compound-specific behavior and study conditions, and should not be generalized across all glucuronides. Sulfates were the least abundant among conjugate classes. This may be due to insewer degradation or hydrolysis of these metabolites, consistent with reports of desulfation in bioactive wastewater environments (Al-Horani and Desai 2010; Gao et al. 2018; Scherer et al. 2025). Alternatively, sulfation may occur less broadly across xenobiotic classes than glucuronidation or mercapturate formation, producing fewer detectable sulfate conjugates overall (Jancova et al. 2010).

Metabolite distributions varied across the study area. Untargeted profiling identified 41 metabolites detected at all sites and a core group of 15 that were detected at every site. Within this group, several MAs derived from volatile organic compounds, including 2CoEMA, 3HPMA, 34HBMA, 2CaEMA, 2CyEMA, 3HMPMA, 2HEMA, 2HPMA, and BzMA, were detected in both untargeted and targeted analyses and showed comparable spatial distributions across sites. Both datasets showed elevated responses in the smaller upstream neighborhood catchments (sites S1–S4) and lower levels in treatment-center influent (S9, S15). Neighborhood sites displayed broader interquartile ranges and higher variability for 3HPMA, 2CaEMA, 2CyEMA, 3HMPMA, and 2HPMA (p < 0.05), which may reflect localized variability associated with traffic and industrial sources. In contrast, treatment centers integrated wastewater from larger, demographically mixed populations and produced more stable concentration profiles. The consistent spatial patterns across ILGA and LC-MS/MS results demonstrate that untargeted Phase II metabolite profiling can reliably indicate community-level exposure gradients, while targeted analysis provides quantitative confirmation within the same chemical domain.

Beyond site-specific variation, spatial trends also differed among conjugate classes. GAs dominated across sites, followed by MAs and SAs, consistent with differences in conjugation pathways and metabolite persistence in wastewater. Glucuronidation is a major Phase II pathway in humans and occurs across a wide spectrum of xenobiotic and endogenous substrates (Chen et al. 2025; Croom 2012). As a result, GAs include metabolites from a larger chemical domain, including pharmaceuticals, environmental pollutants, and dietary constituents, which accounts for their greater abundance in wastewater (Chen et al. 2025; Jancova et al. 2010). Mercapturates, in contrast, derive primarily from reactive electrophiles such as VOCs and other compounds that conjugate with glutathione, forming a narrower subset of environmental metabolites (Hanna and Anders 2019; Xie et al. 2023). Finally, SAs were least abundant, consistent with their polarity and potential susceptibility to microbial desulfation in sewer environments (Al-Horani and Desai 2010; Gao et al. 2018; Scherer et al. 2025). These patterns indicate that GA, MA, and SA distributions in wastewater follow metabolic trends in humans, with glucuronidation occurring across a wider range of substrates and sulfation limited to fewer compounds. Each catchment corresponds to demographically distinct neighborhoods with differing land use and industrial proximity (Holm et al. 2022; Yeager et al. 2021).

Although the sampling framework was designed to include neighborhoods that vary in population size and socioeconomic characteristics, the present study did not directly evaluate associations between these indicators and metabolite levels. The spatial heterogeneity observed here may reflect differences in local emission sources and community characteristics. Future studies that incorporate demographic and disparity datasets will be needed to determine whether elevated VOCm loads coincide with differences in socioeconomic or health vulnerability.

After evaluating several normalization approaches that did not yield conclusive results, comparisons were based on unnormalized responses. Rigorous sampling protocols, identical sample volumes, and analytical reproducibility supported reliable relative interpretation across sites; nevertheless, future work will focus on developing and validating appropriate data correction approaches.

### Source Attribution: Industrial and Environmental Exposures:

Spatial and chemical patterns in the dataset provide insight into the environmental and industrial sources contributing to detected metabolites, especially for Phase II conjugates, which tend to be less studied. Several Phase II parent compounds correspond to Toxic Release Inventory (TRI)-listed chemicals (United States Environmental Protection 2025b), including 1HMPeMA and 4HBeMA (1,3-butadiene), 2CyEMA (acrylonitrile), MCaMA (N,N-dimethylformamide), and MUCA (benzene). These metabolites were consistently detected across most sites, including locations without reported industrial releases in the study area, suggesting contributions from diffuse or unreported sources. The detection of these metabolites in both catchments which are predominately residential and some which are mixed residential and industrial and demonstrates that wastewater integrates signatures from multiple exposure pathways, providing an independent layer of evidence complementary to regulatory emission inventories. Across the untargeted dataset, 112 of the 194 detected VOCm appeared three or more times across sites, representing 79 unique parent compounds, 19 of which are listed in the TRI database. Nine of these (1,3-butadiene, acrylonitrile, butyl acrylate, formaldehyde, methyl acrylate, naphthalene, styrene, toluene, and benzene), have recent documented TRI releases in Jefferson County. 1,3-Buta- diene, produced from petroleum processing, is particularly notable because it is not commonly associated with household sources and was detected in all wastewater samples as MA54 and MA46 (1,3-butadiene), consistent with prior air-monitoring data from Louisville (Mukerjee et al. 2020). Methyl acrylate, while reported to have been released locally, appeared less frequently (25/64 samples), suggesting that wastewater detection depends not only on emission magnitude but also on compound volatility, reactivity, and persistence. Twelve additional TRI-listed parent compounds, including acrolein, glycidol, propylene oxide, acrylamide, and N,N-dimethylformamide, were detected despite no reported releases in Jefferson County. Among these, MCaMA (N,N- dimethylformamide) appeared in every sample (64/64), indicating either unreported emissions or diffuse background use in industrial and laboratory settings. MCaMA’s consistent detection may also reflect greater chemical stability in wastewater than more reactive VOC- derived metabolites. These differences between measured wastewater signals and reported emissions show that wastewater analysis can identify both active and residual contamination not captured by current reporting systems. Detection of airborne VOC-derived metabolites such as 1HMPeMA and 4HBeMA (1,3-butadiene) and 2CyEMA (acrylonitrile) paralleled atmospheric VOC measurements from the same region (Mukerjee et al. 2020), supporting a connection between inhalation exposure and urinary metabolite excretion. The detected Phase II compounds represented a mixture of industrial, consumer, and environmental inputs. Industrial markers such as 1HMPeMA and 4HBeMA (1,3- butadiene), 2CyEMA (acrylonitrile), MCaMA (N,N-dimethylforma- mide), and MUCA (benzene) occur together with consumer and pharmaceutical metabolites such as BzMA (toluene/benzyl alcohol) and AcEMA (acetaminophen), and with natural metabolites including VMA (vanillin) and HBA (hydroxybenzoic acid). This combination shows that wastewater captures exposures arising from routine activities and indoor environments, in addition to ambient and industrial sources. Most of the detected compounds are known human urinary metabolites, although environmental or non-human contributions cannot be completely excluded. The structural specificity of these metabolites to human detoxication pathways indicates that wastewater signals primarily originate from human excretion. When evaluated alongside TRI data and local land-use information, these results show that wastewaterbased exposomics complements emission inventories and expands the capacity of community-scale surveillance to resolve chemical exposures in urban environments.

Wastewater captures composite exposures to both industrial pollutants and consumer chemicals, providing a community-level complement to traditional biomonitoring surveys. The consistent detection of metabolites such as MUCA (benzene), 3HPMA (acrolein), 1HMPeMA and 4HBeMA (1,3-butadiene) indicates widespread population exposure to compounds linked to cardiopulmonary disease, neurological disorders, cancer, and immune dysfunction (Abplanalp et al. 2017; Liao et al. 2022; Riggs et al. 2022; Wan et al. 2024; Wang et al. 2024). These detected metabolites mirror those measured in large-scale human studies such as NHANES (National Center for Health, 2025), confirming the chemical burden observed in wastewater reflects real and biologically relevant exposures. The overlap between wastewater detections and airborne VOC profiles reported for the same region (Mukerjee et al. 2020) further demonstrates that inhaled pollutants are metabolized and excreted in forms traceable through wastewater, effectively linking environmental presence to human uptake. This connection strengthens the interpretation that community-scale wastewater signals provide an integrated proxy of both environmental and behavioral exposures, including inhalation and product use. Because neighborhood-level catchments represent demographically distinct populations (Holm et al. 2022; Yeager et al. 2021), linking chemical profiles to socioeconomic and health indices could reveal whether higher VOCm loads coincide with neighborhoods experiencing greater environmental and/or public health vulnerability. Integrating WBE outputs with datasets such as EPA’s Environmental Justice Screening and Mapping Tool (EJSCREEN) (United States Environmental Protection 2025a) or local health indicators would allow spatial comparison of chemical exposure intensity and population risk.

Collectively, these findings illustrate how wastewater-based exposomics can extend traditional biomonitoring by adding spatial resolution and community context. By coupling high-frequency environmental sampling with established biomarker interpretation frameworks, WBE provides an early-warning and prioritization tool for identifying communities with disproportionate chemical burdens.

### Limitations:

Interpretation of these results is constrained by several factors inherent to wastewater analysis. Temporal coverage was limited to four sampling periods, which may not fully capture seasonal or short-term variability. Some metabolites, particularly sulfates and other labile conjugates, may degrade during transport or storage, leading to underestimation of their true abundance. In addition, many Phase II conjugates, including glucuronides and sulfates, may originate from non-human sources such as pet waste, urban wildlife, or agricultural runoff rather than exclusively from human excretion. Beyond degradation, microbial activity within sewer biofilms may also transform or synthesize conjugated metabolites, creating the potential for altered signals or false-positive attribution for certain human biomarkers. The ILGA library, although extensive, does not encompass the full range of potentially conjugated metabolites, and annotation confidence remains limited by the availability of standards. The current chromatographic gradient was not optimized for larger, more hydrophobic PAH metabolites, which were likely retained on the column and not fully eluted, limiting their detection in the untargeted workflow. Dedicated methods will be needed to improve recovery of these metabolites. In addition, because the targeted assay applied here was an existing validated method for mercapturates and related VOCm biomarkers, direct cross-platform confirmation was limited to a small subset of shared metabolites and did not include glucuronides or sulfates. Addressing this limitation will require expanded targeted methods for these conjugate classes. Future studies should refine normalization to improve quantitative interpretation. Broader temporal sampling would help distinguish persistent exposure patterns from transient events. Expansion of the ILGA library to include additional Phase II conjugates and validation across different wastewater systems will extend the analytical reach of this framework. Integrating demographic, emission, and other environmental monitoring and health datasets could link community-scale exposures with potential biological outcomes. Continued alignment of untargeted discovery with targeted quantitation will enable more comprehensive and interpretable assessments of population-level chemical exposures.

## 5. Conclusions

This study establishes an integrated wastewater exposomics framework combining untargeted Phase II metabolite profiling with targeted LC–MS/MS for community-level exposure surveillance across neighborhood and treatment-scale sewersheds. This approach extends wastewater-based epidemiology beyond limited target panels, enabling broader monitoring of human exposure biomarkers. By linking wastewater measurements to biomarker-based interpretation, it complements traditional monitoring while improving community-scale resolution. Further refinement of normalization strategies, metabolite libraries, and data integration will enhance its utility for population-level exposure assessment.

## Supplementary Material

Sup Data 1

Sup Data 2

## Figures and Tables

**Table 1:** Detection consistency of Phase II metabolites across wastewater sampling sites and time points.

Name	Group	Proposed parent	Metabolite name	Reps
MA19	Aliphatic aldehydes	4-Hydroxy-2-hexenal	N-Acetyl-S-[1-(2-oxoethyl)-2-hydroxybutyl]-L-cysteine	1+
MA23		4-Hydroxy-2-octenal	N-Acetyl-S-[1-(carboxymethyl)-2-hydroxyhexyl]-L-cysteine	1+
MA36		Acrolein	N-Acetyl-S-(2-carboxyethyl)-L-cysteine	1+
MA51		2-Pentenal	N-Acetyl-S-(1-ethyl-3-hydroxypropyl)-L-cysteine	1+
MA81		2-Hexenal	N-Acetyl-S-[(1S)-1-(2-oxoethyl)butyl]-L-cysteine	1+
MA66		Cinnamaldehyde	N-Acetyl-S-(3-hydroxy-1-phenylpropyl)-L-cysteine	3+
MA58	Benzene/Mono aromatics	Hydroquinone	N-Acetyl-S-(2,5-dihydroxyphenyl)-L-cysteine	1+
MA56	Epoxide	Glycidol	N-Acetyl-S-(2,3-dihydroxypropyl)-L-cysteine	1+
MA47	Halogenated aliphatics	Halogenated butene	N-Acetyl-S-(1,2-dihydroxybutyl)-L-cysteine	1+
MA49		Halogenated butene	N-Acetyl-S-(1,2-dihydroxybutyl)-L-cysteine	3+
MA2	Monoterpenes and derivatives	Limonene	N-Acetyl-S-(limonene-8,9-diol)-L-cysteine	1+
MA4		Limonene	N-Acetyl-S-(limonene-1,2-diol)-L-cysteine	1+
MA32	Other aliphatics	N,N-dimethylformamide	N-Acetyl-S-(N-methylcarbamoyl)-L-cysteine	3+
MA46		1,3-Butadiene	N-Acetyl-S-(2-hydroxy-3-buten-1-yl)-L-cysteine	3+
MA54		1,3-Butadiene	N-Acetyl-S-(2,3,4-trihydroxybutyl)-L-cysteine	3+
MA64		Butyl acrylate	N-Acetyl-S-(3-butoxy-3-oxopropyl)-L-cysteine	1+
MA76		2-Methylpropyl carbamic acid	N-Acetyl-(2-methylpropylcarbamate)-L-cysteine	1+
MA73		Benomyl	N-Acetyl-S-(butylcarbamate)-L-cysteine	1+
MA30	Aromatics	Acetaminophen	N-Acetyl-S-[5-(acetylamino)-2-hydroxyphenyl]-L-cysteine	3+
MA71	Polycyclic aromatic hydrocarbons	Benzo[a]pyrene	N-Acetyl-S-(7,8,9,10-tetrahydro-8,9,10-trihydroxybenzo[a]pyren-7-yl)-L- cysteine	1+
GA39	Aliphatic aldehydes	4-Hydroxy-2-heptenal	2-Heptenal-4-glucuronide	1+
GA92		4-Hydroxy-2-octenal	4-Hydroxy-octenylcarboxylic acid glucuronide	3+
GA10	Benzene/Mono aromatics	1-(4-Methylphenyl)ethanol	1-(4-Methylphenyl)ethyl β-D-glucopyranosiduronic acid	3+
GA16		Phenylpropanoic acid	1-benzenepropanoate β-D-glucopyranuronic acid	3+
GA19		4-Phenyl-3-buten-2-ol	1-Methyl-3-phenyl-2-propen-1-yl β-D-glucopyranosiduronic acid	1+
GA35		Propenylguaethol	2-Ethoxy-5-(1-propen-1-yl)phenyl β-D-glucopyranosiduronic acid	3+
GA54		2-Methyl-5-phenylpentanol	2-Methyl-5-phenylpentyl β-D-glucopyranosiduronic acid	3+
GA3	Bile acids	Glycocholic acid	Glycocholic acid 3-glucuronide	1+
GA100	Dietary phenolics and flavonoids	Dihydrocaffeic acid	5-(2-Carboxyethyl)-2-hydroxyphenyl β-D-glucopyranosiduronic acid	1+
GA87		Vanillin	4-Formyl-2-methoxyphenyl β-D-glucopyranosiduronic acid	3+
GA88		Vanillin	4-Formyl-2-methoxyphenyl β-D-glucopyranosiduronic acid	3+
GA31	Halogenated aromatics	2,6-Dichlorophenol	2,6-Dichlorophenyl β-D-glucopyranosiduronic acid	3+
GA8		2-Fluorobenzoic acid	1-(2-fluorobenzoate) β-D-glucopyranuronic acid	1+
GA28	Other aliphatics	Glycerol	2,3-Dihydroxypropyl α-D-glucopyranosiduronic acid	1+
GA95		4-Oxo-2-heptenoic acid	4-Oxo-2-heptenoic acid glucuronide	1+
GA2		2,4-Dimethyl-3-cyclohexene-1- methanol	(2,4-Dimethyl-3-cyclohexen-1-yl)methyl β-D-glucopyranosiduronic acid	1+
GA13	Polycyclic aromatic hydrocarbons	Naphthalene	1,2-Dihydro-1-hydroxy-2-naphthalenyl β-D-glucopyranosiduronic acid	1+
GA20		Naphthalene	1-Naphthol glucuronide	1+
SA18	Aromatics	Acetylphenol	4-Acetylphenol sulfate	1+
SA39		Acetaminophen	Acetaminophen sulfate	3+
SA55		Coumaric acid	p-Coumaric acid sulfate	1+

This table lists the 41 metabolites observed in at least one sample from every site, along with their class, parent group, proposed parent, and an annotated metabolite name.

**Table 2:** Volatile organic compound (VOC) derived metabolites detected by untargeted analysis and their overlap with the Comprehensive VOC and TA (CVT) assay coverage.

Name	Metabolite name	Acronym	Proposed parent
MA38	N-Acetyl-S-(3,4-dihydroxybutyl)- L-cysteine	34HBMA	1,3-Butadiene
MA15	N-Acetyl-S-(3-hydroxypropyl)-L- cysteine	3HPMA	Acrolein
MA36	N-Acetyl-S-(2-carboxyethyl)-L- cysteine	2CoEMA	Acrolein
MA29	N-Acetyl-S-(2-carbamoylethyl)-L- cysteine	2CaEMA	Acrylamide
MA40	N-Acetyl-S-(2-carbamoyl-2- hydroxyethyl)-L-cysteine	2CaHEMA	Acrylamide
MA37	N-Acetyl-S-(2-cyanoethyl)-L- cysteine	2CyEMA	Acrylonitrile
MA42	N-Acetyl-S-(3-hydroxy-1- methylpropyl)-L-cysteine	3HMPMA	Crotonaldehyde
MA41	N-Acetyl-S-(2-hydroxyethyl)-L- cysteine	2HEMA	Ethylene oxide
MA56	N-Acetyl-S-(2,3- dihydroxypropyl)-L-cysteine	23HPMA	Glycidol
MA44	N-acetyl-S-(2-hydroxy-3-methyl- 3-buten-1-yl)-L-cysteine	2HMBeMA	Isoprene
MA70	N-Acetyl-S-(4-hydroxy-2-methyl- 2-buten-1-yl)-L-cysteine	4HMBeMA	Isoprene
MA32	N-Acetyl-S-(N-methylcarbamoyl)- L-cysteine	MCaMA	N,N- dimethylformamide
MA11	N-Acetyl-S-(2-hydroxypropyl)-L- cysteine	2HPMA	Propylene oxide
MA34	N-Acetyl-S-benzyl-L-cysteine	BzMA	Toluene

This table lists 14 metabolites of known volatile organic compounds (VOCs) identified using our untargeted high-resolution liquid chromatography-mass spectrometry workflow. Metabolites marked “Yes” are also measurable using the Comprehensive VOC and TA (CVT) assay, a validated targeted method that quantifies 35 urinary biomarkers of exposure, including VOC metabolites and tobacco alkaloids. (Srivastava et al., 2025).

## Data Availability

The data that support the findings of this study are available on request from the corresponding authors.

## Appendix A. Supplementary data

Supplementary data to this article can be found online at https://doi.org/10.1016/j.envint.2026.110293.
