# Supplementary material for: A unified liquid chromatography-mass spectrometric approach combining targeted and untargeted analyses for community exposure profiling in wastewater
Source: Environ Int. Author manuscript; Available in PMC 2026 Jul 6. (PMC13334828; doi:10.1016/j.envint.2026.110293)
Supplement: Sup Data 2 [file NIHMS2181951-supplement-Sup_Data_2.docx]

**Table of Contents**

[**Table S1**. Demographics of studied wastewater sample locations (N = 16). 3](#_Toc226734293)

[**Table S2**. Product numbers for the VOC and TA analytes and their corresponding internal standards, and solvents used for the preparation of stocks. 5](#_Toc226734294)

[**Table S3.** Method limit of detection (LOD) and limit of quantitation (LOQ) for each analyte. 7](#_Toc226734295)

[**Table S4.** Method precision expressed as coefficient of variation (%CV). Values calculated from five replicate QC sample runs. 8](#_Toc226734296)

[**Table. S5**. <<Excel file>>. List of all phase II metabolites and their proposed parent compounds detected in wastewater samples using the untargeted LC-MS/QTOF method. 8](#_Toc226734297)

[**Table. S6**. <<Excel file>>. List of all phase II metabolites and their proposed parent compounds detected in wastewater samples using the untargeted LC-MS/QTOF method. 8](#_Toc226734298)

[**Table. S7**. VOC-derived metabolites detected in wastewater samples by the CVT targeted LC-MS/MS assay. This table lists 14 urinary metabolites of known volatile organic compounds (VOCs) identified using our untargeted high-resolution LC-MS workflow. Metabolites marked “*” are phase I metabolites. Metabolites marked with “**^U^**” were also detected using the untargeted profiling. 9](#_Toc226734299)

[**Table. S8**. Concentration ranges and median values of VOC-derived metabolites in wastewater samples (N = 64). Measured by the CVT targeted LC-MS/MS assay. 10](#_Toc226734300)

[**Table. S9**. Comparison of interquartile concentration ranges of volatile organic compound metabolites between neighborhood catchment locations and wastewater treatment center sites. 11](#_Toc226734301)

[**Table. S10**. <<Excel file>>. Concentrations of biomarkers used for normalization (cotinine, 5-HIAA and PMMoV). 11](#_Toc226734302)

[**Figure S1**. Network representation of core Phase II metabolites by conjugate class, showing spatial patterns in metabolite intensity and variation across the sampling system. Each panel corresponds to one metabolite class: (A) mercapturic acids (MAs), (B) glucuronides (GAs), and (C) sulfates (SA). Metabolites (purple) are connected to sampling locations where they showed above-average (red edges, z-score > 1) or below-average (blue edges, z-score < –1) normalized abundance. Manholes are shown in orange and treatment plants in green. 12](#_Toc226734303)

[**Figure S2**. Comparison of variation (expressed as %CV) between normalization modes. Abbreviations: COT – cotinine, 5HIAA - 5-hydroxyindoleacetic acid , PMMOV - pepper mild mottle virus. 13](#_Toc226734304)

[**Figure S3**. Comparison of variation (expressed as ΔCV) between normalization modes. Abbreviations: COT – cotinine, 5HIAA - 5-hydroxyindoleacetic acid , PMMOV - pepper mild mottle virus. 13](#_Toc226734305)

[**Figure S4**. 2HPMA concentration normalized by (A) PMMoV and (B) population size of the studied sewershed. N=64 14](#_Toc226734306)

[**Figure S5**. 2MHA concentration normalized by (A) PMMoV and (B) population size of the studied sewershed. N=64 14](#_Toc226734307)

[**Figure S6**. 3HPMA concentration normalized by (A) PMMoV and (B) population size of the studied sewershed. N=64 15](#_Toc226734308)

[**Figure S7**. 34MHA concentration normalized by (A) PMMoV and (B) population size of the studied sewershed. N=64 15](#_Toc226734309)

[**Figure S8**. AAMA concentration normalized by (A) PMMoV and (B) population size of the studied sewershed. N=64 16](#_Toc226734310)

[**Figure S9**. BMA concentration normalized by (A) PMMoV and (B) population size of the studied sewershed. N=64 16](#_Toc226734311)

[**Figure S10**. 1PMA concentration normalized by (A) PMMoV and (B) population size of the studied sewershed. N=64 17](#_Toc226734312)

[**Figure S11**. 2CoEMA concentration normalized by (A) PMMoV and (B) population size of the studied sewershed. N=64 17](#_Toc226734313)

[**Figure S12**. 2CyEMA concentration normalized by (A) PMMoV and (B) population size of the studied sewershed. N=64 18](#_Toc226734314)

[**Figure S13**. 34HBMA concentration normalized by (A) PMMoV and (B) population size of the studied sewershed. N=64 18](#_Toc226734315)

[**Figure S14**. 2HEMA concentration normalized by (A) PMMoV and (B) population size of the studied sewershed. N=64 19](#_Toc226734316)

[**Figure S15**. HMPMA concentration normalized by (A) PMMoV and (B) population size of the studied sewershed. N=64 19](#_Toc226734317)

[**Figure S16**. MADA concentration normalized by (A) PMMoV and (B) population size of the studied sewershed. N=64 20](#_Toc226734318)

[**Figure S17**. MUCA concentration normalized by (A) PMMoV and (B) population size of the studied sewershed. N=64 20](#_Toc226734319)

[**Figure S18**. PHEMA concentration normalized by (A) PMMoV and (B) population size of the studied sewershed. N=64 21](#_Toc226734320)

# **Table S1**. Demographics of studied wastewater sample locations (N = 16).

| Map ID/Site Number | Site | Mean median household income ($) | Population | Site area (km^2^) | Treatment plant | Combined sewer | Percent white | Percent Black or African American | Percent Hispanic or Latino |
| --- | --- | --- | --- | --- | --- | --- | --- | --- | --- |
| 16 | MFWQTC | 54,138.27 | 349850 | 280 |  | Yes | 68 | 25 | 4 |
| 5 | DRGWQTC | 53,576.87 | 295910 | 332 |  | No | 72 | 21 | 7 |
| 8 | CCWQTC | 76,605.69 | 55928 | 80 |  | No | 82 | 12 | 4 |
| 10 | FFWQTP | 113,699.07 | 32460 | 88 |  | No | 87 | 8 | 3 |
| 11 | HCWQTP | 106,769.23 | 31269 | 67 |  | No | 75 | 14 | 4 |
| 1 | Shawnee Park CSO189 | 27,694.54 | 10739 | 5 | MFWQTC | Yes | 9 | 88 | 1 |
| 2 | 34th Street PS | 27,446.22 | 7820 | 5 | MFWQTC | Yes | 68 | 26 | 3 |
| 12 | MH40870 Muddy Forks PS | 103,303.86 | 11203 | 12 | MFWQTC | Yes | 92 | 3 | 2 |
| 3 | MH32985 Wood Road & Terry Road | 45,895.09 | 35956 | 28 | DRGWQTC | No | 59 | 37 | 4 |
| 4 | MH09837 Ashby Lane & Mill Creek | 51,656.41 | 25073 | 21 | DRGWQTC | No | 83 | 12 | 5 |
| 13 | MH08915A CSO140 | 77,842.30 | 99061 | 80 | MFWQTC | Yes | 87 | 7 | 4 |
| 15 | MH23290 W. Indian Trail | 53,542.20 | 73666 | 55 | DRGWQTC | No | 63 | 28 | 9 |
| 7 | MH57769 Pineland Drive & Oakmont Dr. | 61,836.52 | 46659 | 37 | DRGWQTC | No | 75 | 18 | 8 |
| 6 | MH57350 Preston & South Park | 63,642.42 | 22437 | 23 | DRGWQTC | No | 80 | 13 | 5 |
| 14 | MH71910 CSO146 | 49,031.13 | 8071 | 3 | MFWQTC | Yes | 90 | 6 | 3 |
| 9 | MH70101 15th & Wilson | 24,084.47 | 20832 | 12 | MFWQTC | Yes | 61 | 32 | 5 |

# **Table S2**. Product numbers for the VOC and TA analytes and their corresponding internal standards, and solvents used for the preparation of stocks.

| # | Analyte | | Internal standard | | Stock solvent |
| --- | --- | --- | --- | --- | --- |
|  | Acronym | Product number | Acronym | Product number |  |
| 1 | 2CoEMA | TRC-A168045 | 2CoEMA-d_3_ | TRC-A168047 | water |
| 2 | 3HPMA | TRC-A179075 | 3HPMA-d_3_ | TRC-A179077 | water |
| 3 | 2CaHEMA | TRC-A179130 | 2CaHEMA-d_3_ | TRC-A179132 | water |
| 4 | 2CaEMA | TRC-A171870 | 2CaEMA-d_4_ | CDN Isotopes-D-6589 | water |
| 5 | 2HEMA | TRC-A178911 | 2HEMA-d_4_ | TRC-A178912 | water |
| 6 | 2CyEMA | TRC-A172083 | 2CyEMA-d_3_ | TRC-A172084 | water |
| 7 | 1PMA | TRC-A168035 | 1PMA-d_7_ | TRC-A168037 | water |
| 8 | 34HBMA | TRC-A173710 | 34HBMA-d_7_ | TRC-A173712 | water |
| 9 | HMPeMA | TRC-A179005 | HMPeMA-d_6_ | TRC-A179007 | water |
| 10 | 2HBeMA | TRC-A178645 | 2HBeMA-d_3_ | TRC-A178647 | 50% MeOH |
| 11 | HPMMA | TRC-A179125 | HPMMA-d_3_ | TRC-A179127 | water |
| 12 | MCaMA | TRC-A186625 | MCaMA-d_3_ | TRC-A186627 | water |
| 13 | 23HPMA | TRC-D965260 | 23HPMA-d_5_ | TRC-D965261 | water |
| 14 | 1HMMPeMA | TRC-A179020 | 1HMMPeMA-d_3_ | TRC-A179022 | MeOH |
| 15 | 3HMMPeMA | TRC-A179035 | 1HMMPeMA-d_3_ | TRC-A179022 | MeOH |
| 16 | 2HPMA | TRC-A179060 | 2HPMA-d_3_ | TRC-A179062 | water |
| 17 | 122CVMA | TRC-A189580 | 122CVMA-d_3_ | TRC-A189582 | MeOH |
| 18 | 12CVMA | TRC-A173585 | 12CVMA-^13^C,d_3_ | TRC-A173587 | MeOH |
| 19 | 22DCVMA | TRC-A173580 | 22DCVMA-^13^C,d_3_ | TRC-A173582 | MeOH |
| 20 | MUCA | Sigma-M90003 | MUCA-^13^C_6_ | Sigma-900846 | EtOH |
| 21 | Pre-PhMA | ASI Chemicals-  79-5544-01 | Pre-PhMA-^13^C_3_,^15^N | ASI Chemicals-79-5760-01 | 10mM ammonium bicarbonate in water |
| 22 | PhMA | TRC-P335600 | PhMA-d5 | TRC-P335605 | 50% MeOH |
| 23 | PhGA | TRC-P327500 | PhGA-d5 | CDN Isotopes-D-7205 | water |
| 24 | MADA | Sigma-M2101 | MADA-d5 | TRC-M162527 | 50% MeOH |
| 25 | 2HPhEMA | TRC-A179025 | 2HPhEMA-d_3_ | TRC-A179027 | MeOH |
| 26 | BzMA | TRC-A168428 | BzMA-d_3_ | TRC-A168427 | MeOH |
| 27 | 2MHA | Sigma-328006 | 2MHA-d_7_ | CDN Isotopes-D-7202 | 50% MeOH |
| 28 | 3MHA +  4MHA | Sigma-328014,  Sigma-328022 | 3MHA-d_7_ +  4MHA-d_7_ | CDN Isotopes-D-7203,  CDN Isotopes-D-7204 | 50% MeOH |
| 29 | 34MPhMA +  24MPhMA +  25MPhMA | TRC-A173755,  TRC-A173750,  TRC-A173770 | 34MPhMA-d_3_ + 24MPhMA-d_3_ + 25MPhMA-d_3_ | TRC-A173757,  TRC-A173752,  TRC-A173772 | MeOH |

# **Table S3.** Method limit of detection (LOD) and limit of quantitation (LOQ) for each analyte.

| Metabolite | r^2^ | Method LOD picograms/mL | Method LOQ picograms/mL |
| --- | --- | --- | --- |
| MUCA | 0.999 | 5.43 | 10.85 |
| 2CoEMA | 0.996 | 2.71 | 10.85 |
| 3HMPMA | 0.999 | 0.07 | 0.54 |
| MADA | 0.996 | 0.33 | 2.71 |
| 2CyMA | 0.995 | 0.33 | 2.17 |
| 2MHA | 0.997 | 1.08 | 4.35 |
| 34MHA | 0.995 | 0.28 | 2.17 |
| 1PMA | 0.999 | 0.07 | 0.28 |
| 34HBMA | 0.998 | 0.28 | 1.08 |
| 2CaEMA | 0.998 | 0.28 | 0.54 |
| 2HEMA | 0.998 | 0.22 | 0.43 |
| 3HPMA | 0.997 | 0.08 | 0.33 |
| 2HPMA | 0.998 | 0.14 | 0.54 |
| BzMA | 0.995 | 0.03 | 0.22 |
| 4HMBeMA | 0.997 | 0.54 | 1.08 |
| 2HPhEMA | 0.998 | 0.01 | 0.54 |

# **Table S4.** Method precision expressed as coefficient of variation (%CV). Values calculated from five replicate QC sample runs.

| Acronym | %CV |
| --- | --- |
| Acrolein\|\|3HPMA | 2 |
| Acrolein\|\|2CoEMA | 15 |
| Acrylamide\|\|2CaMA | 3 |
| Acrylamide\|\|34HBMA | 2 |
| Acrylonitrile\|\|2CyEMA | 4 |
| Benzene\|\|MUCA | 4 |
| Bromopropane\|\|1PMA | 3 |
| Crotonaldehyde\|\|3HMPMA | 1 |
| Dibromoethane\|\|2HEMA | 10 |
| Propylene oxide\|\|2HPMA | 2 |
| Propylene oxide\|\| 2HPhEMA | 14 |
| Styrene\|\|MADA | 4 |
| Toluene, Benzyl alcohol\|\|BzMA | 4 |
| Xylene\|\|2MHA | 28 |
| Xylene\|\|34MHA | 3 |

# **Table. S5**. <<Excel file>>. List of all phase II metabolites and their proposed parent compounds detected in wastewater samples using the untargeted LC-MS/QTOF method.

# **Table. S6**. <<Excel file>>. List of all phase II metabolites and their proposed parent compounds detected in wastewater samples using the untargeted LC-MS/QTOF method.

# **Table. S7**. VOC-derived metabolites detected in wastewater samples by the CVT targeted LC-MS/MS assay. This table lists 14 urinary metabolites of known volatile organic compounds (VOCs) identified using our untargeted high-resolution LC-MS workflow. Metabolites marked “*” are phase I metabolites. Metabolites marked with “**^U^**” were also detected using the untargeted profiling.

| Metabolite name | Acronym | Proposed Parent |
| --- | --- | --- |
| N-Acetyl-S-(2-carboxyethyl)-L-cysteine | 2CoEMA **^U^** | Acrolein |
| N-Acetyl-S-(3-hydroxypropyl)-L-cysteine | 3HPMA **^U^** | Acrolein |
| N-Acetyl-S-(3,4-dihydroxybutyl)-L-cysteine | 34HBMA **^U^** | Acrylamide |
| N-Acetyl-S-(2-carbamoylethyl)-L-cysteine | 2CaEMA **^U^** | Acrylamide |
| N-Acetyl-S-(2-cyanoethyl)-L-cysteine | 2CyEMA **^U^** | Acrylonitrile |
| trans,trans-muconic acid* | MUCA | Benzene |
| N-Acetyl-S-(propyl)-L-cysteine | 1PMA | Bromopropane |
| N-Acetyl-S-(3-hydroxy-1-methylpropyl)-L-cysteine | 3HMPMA **^U^** | Crotonaldehyde |
| N-acetyl-S-(2-hydroxyethyl)-L-cysteine | 2HEMA **^U^** | Dibromoethane |
| N-Acetyl-S-(2-hydroxypropyl)-L-cysteine | 2HPMA **^U^** | Propylene oxide |
| N-Acetyl-S-(2-hydroxy-phenyl)-L-cysteine | 2HPhEMA | Propylene oxide |
| Mandelic acid* | MADA | Styrene |
| N-Acetyl-S-benzyl-L-cysteine | BzMA **^U^** | Toluene, Benzyl alcohol |
| 2-Methylhippuric acid | 2MHA | Xylene |
| 3- and 4-Methylhippuric acids | 3MHA+4MHA | Xylene |

# **Table. S8**. Concentration ranges and median values of VOC-derived metabolites in wastewater samples (N = 64). Measured by the CVT targeted LC-MS/MS assay.

| Parent compound | Metabolite name | Acronym | Concentration range (ng/mL) | Median (ng/mL) |
| --- | --- | --- | --- | --- |
| Acrolein | N-Acetyl-S-(3-hydroxypropyl)-L-cysteine | 3HPMA | 0.03 – 0.99 | 0.37 |
| Acrolein | N-Acetyl-S-(2-carboxyethyl)-L-cysteine | 2CoEMA | 0.02 – 8.13 | 0.17 |
| Acrylamide | N-Acetyl-S-(2-carbamoylethyl)-L-cysteine | 2CaEMA | 0.01 – 0.26 | 0.08 |
| Acrylamide | N-Acetyl-S-(3,4-dihydroxybutyl)-L-cysteine | 34HBMA | 0.01 – 2.23 | 0.07 |
| Acrylonitrile | N-Acetyl-S-(2-cyanoethyl)-L-cysteine | 2CyEMA | 0.01 – 0.23 | 0.04 |
| Benzene | trans,trans-Muconic acid | MUCA | 0.04 – 5.38 | 1.40 |
| Bromopropane | N-Acetyl-S-propyl-L-cysteine | 1PMA | 0.01 – 0.05 | 0.01 |
| Crotonaldehyde | N-Acetyl-S-(3-hydroxy-1-methylpropyl)-L-cysteine | 3HMPMA | 0.01 – 0.59 | 0.19 |
| Dibromoethane | N-Acetyl-S-(2-hydroxyethyl)-L-cysteine | 2HEMA | 0.01 – 0.01 | 0.01 |
| Propylene oxide | N-Acetyl-S-(2-hydroxypropyl)-L-cysteine | 2HPMA | 0.01 – 0.12 | 0.05 |
| Propylene oxide | N-Acetyl-S-(2-hydroxy-phenyl)-L-cysteine | 2HPhEMA | 0.01 – 0.01 | 0.01 |
| Styrene | Mandelic acid | MADA | 0.02 – 1.53 | 0.58 |
| Toluene/benzyl alcohol | N-Acetyl-S-benzyl-L-cysteine | BzMA | 0.01 – 0.03 | 0.01 |
| Xylene | 2-Methylhippuric acid | 2MHA | 0.01 – 0.29 | 0.05 |
| Xylene | 3- and 4-Methylhippuric acids | 3MHA+4MHA | 0.01 – 0.20 | 0.04 |

# **Table. S9**. Comparison of interquartile concentration ranges of volatile organic compound metabolites between neighborhood catchment locations and wastewater treatment center sites.

| Parent compound | Acronym | Average IQR  neighborhood site | Average IQR  treatment center site | p-value |
| --- | --- | --- | --- | --- |
| Acrolein | 3HPMA | 0.22 | 0.09 | **0.006*** |
| Acrolein | 2CoEMA | 0.34 | 0.26 | 0.237 |
| Acrylamide | 2CaEMA | 0.27 | 0.10 | **0.005*** |
| Acrylamide | 34HBMA | 0.45 | 0.39 | 0.404 |
| Acrylonitrile | 2CyEMA | 0.20 | 0.11 | **0.043*** |
| Benzene | MUCA | 0.23 | 0.17 | 0.104 |
| Bromopropane | 1PMA | 0.27 | 0.16 | 0.076 |
| Crotonaldehyde | 3HMPMA | 0.22 | 0.11 | **0.036*** |
| Dibromoethane | 2HEMA | 0.27 | 0.17 | 0.094 |
| Propylene oxide | 2HPMA | 0.26 | 0.09 | **0.005*** |
| Propylene oxide | 2HPhEMA | 0.20 | 0.15 | 0.255 |
| Styrene | MADA | 0.17 | 0.11 | 0.138 |
| Toluene/benzyl alcohol | BzMA | 0.23 | 0.10 | **0.020*** |
| Xylene | 2MHA | 0.21 | 0.18 | 0.288 |
| Xylene | 3MHA+4MHA | 0.27 | 0.30 | 0.378 |

# **Table. S10**. <<Excel file>>. Concentrations of biomarkers used for normalization (cotinine, 5-HIAA and PMMoV).

# **Figure S1**. Network representation of core Phase II metabolites by conjugate class, showing spatial patterns in metabolite intensity and variation across the sampling system. Each panel corresponds to one metabolite class: (A) mercapturic acids (MAs), (B) glucuronides (GAs), and (C) sulfates (SA). Metabolites (purple) are connected to sampling locations where they showed above-average (red edges, z-score > 1) or below-average (blue edges, z-score < –1) normalized abundance. Manholes are shown in orange and treatment plants in green.

# **Figure S2**. Comparison of variation (expressed as %CV) between normalization modes. Abbreviations: COT – cotinine, 5HIAA - 5-hydroxyindoleacetic acid , PMMOV - pepper mild mottle virus.


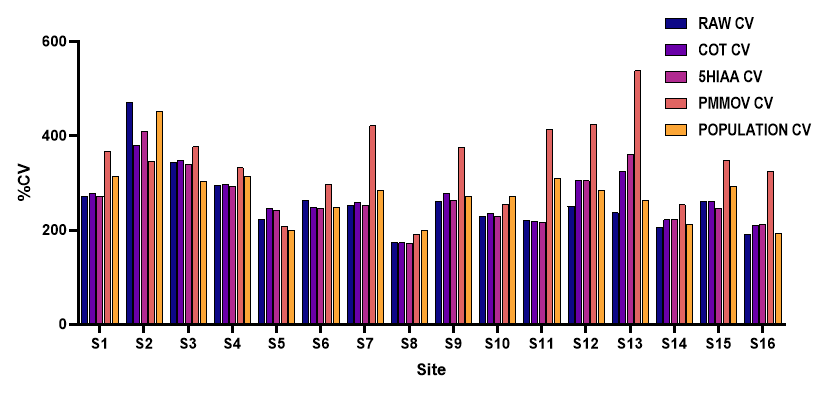


# **Figure S3**. Comparison of variation (expressed as ΔCV) between normalization modes. Abbreviations: COT – cotinine, 5HIAA - 5-hydroxyindoleacetic acid , PMMOV - pepper mild mottle virus.


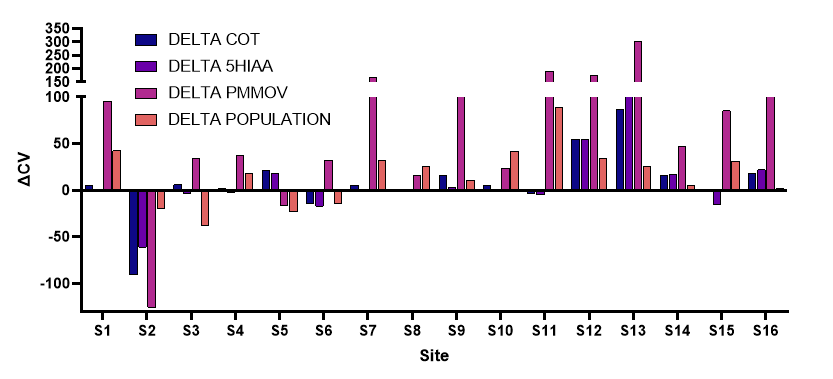


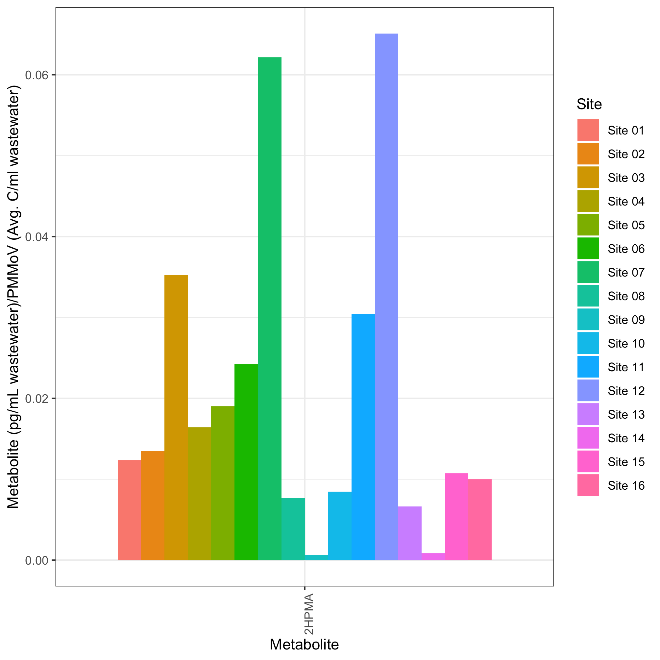

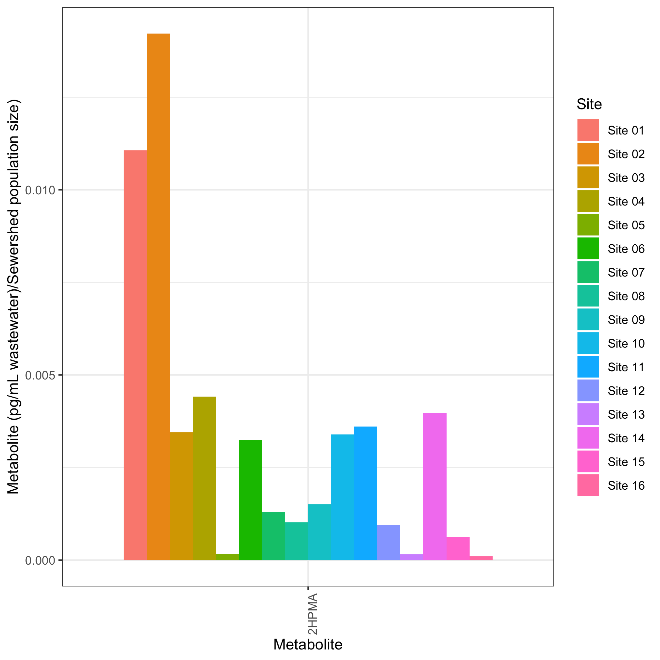


A B

# **Figure S4**. 2HPMA concentration normalized by (A) PMMoV and (B) population size of the studied sewershed. N=64


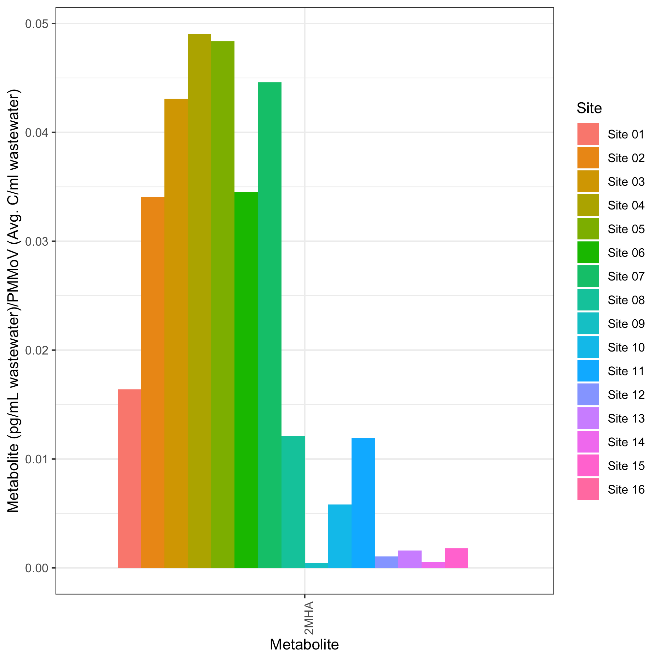

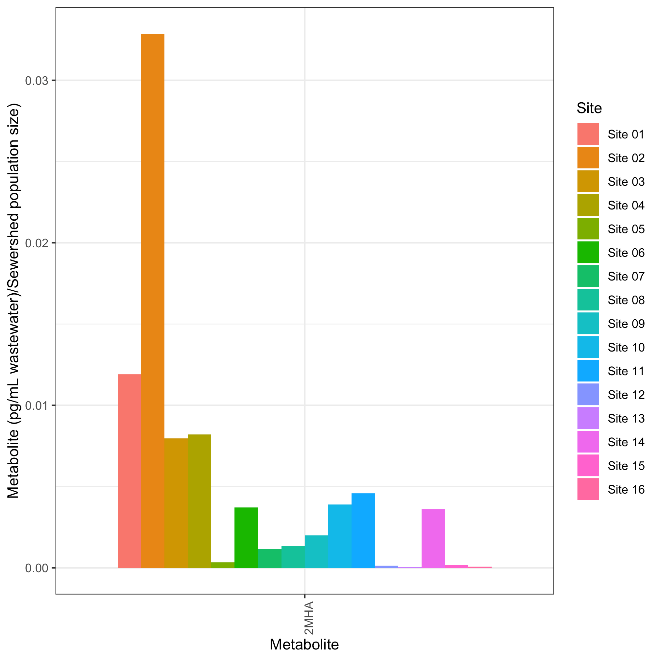


A B

# **Figure S5**. 2MHA concentration normalized by (A) PMMoV and (B) population size of the studied sewershed. N=64


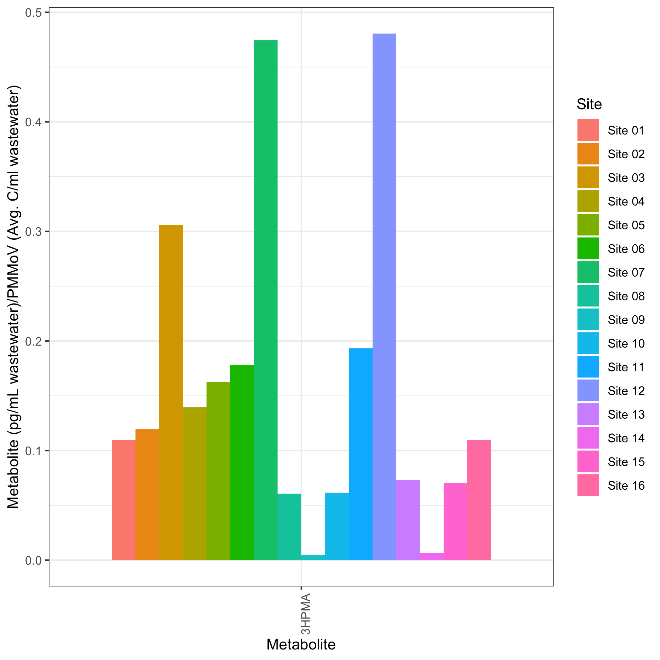

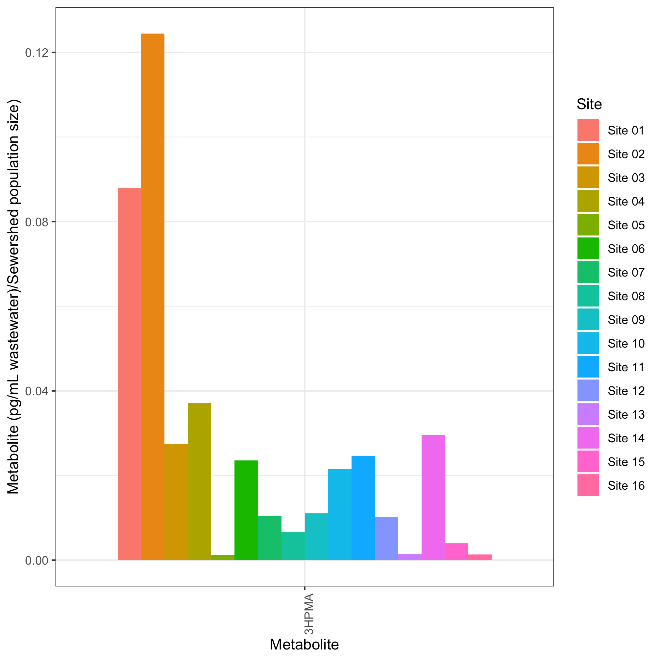


A B

# **Figure S6**. 3HPMA concentration normalized by (A) PMMoV and (B) population size of the studied sewershed. N=64


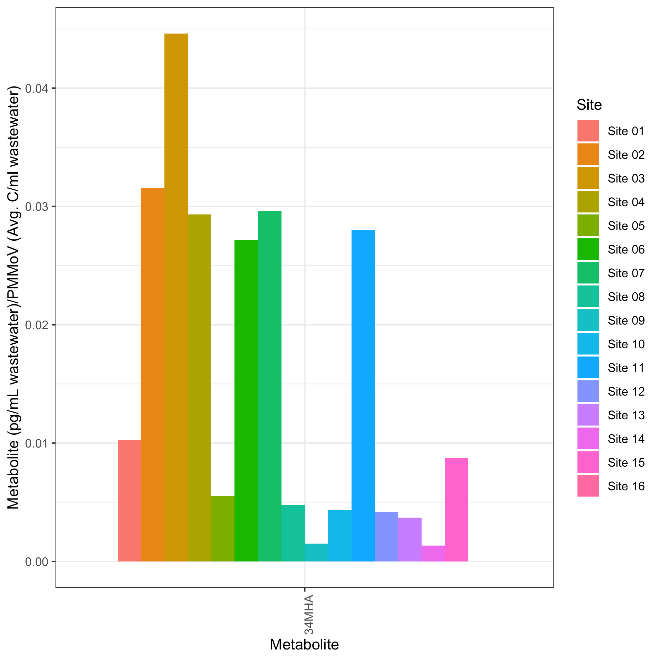

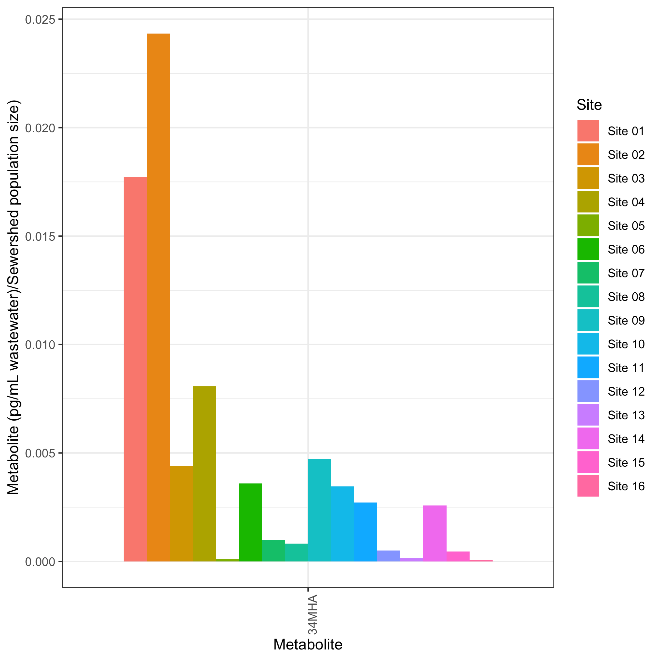


A B

# **Figure S7**. 34MHA concentration normalized by (A) PMMoV and (B) population size of the studied sewershed. N=64


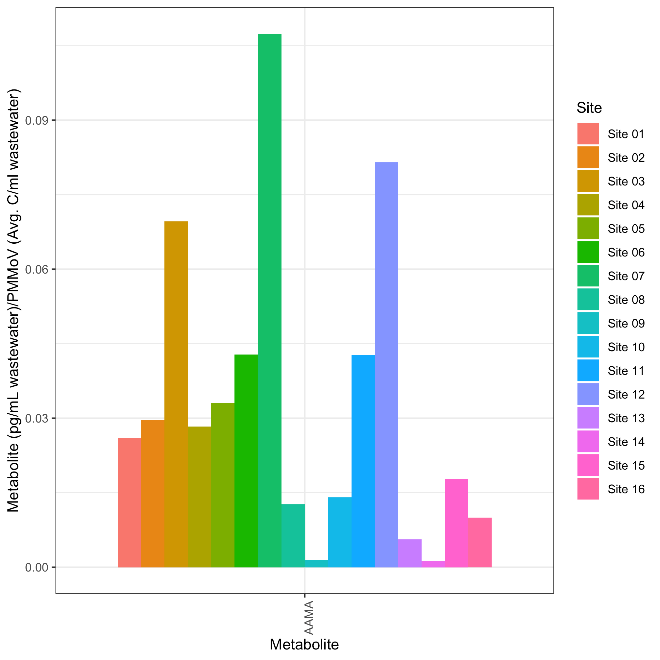

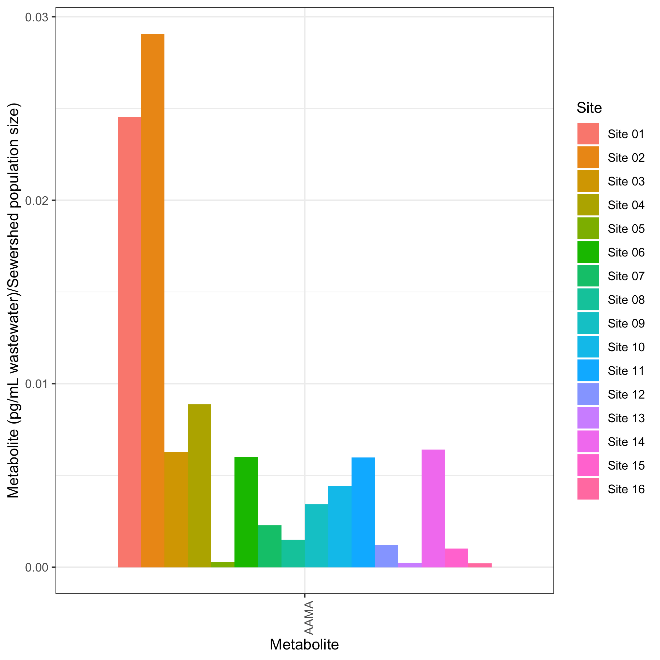


A B

# **Figure S8**. AAMA concentration normalized by (A) PMMoV and (B) population size of the studied sewershed. N=64


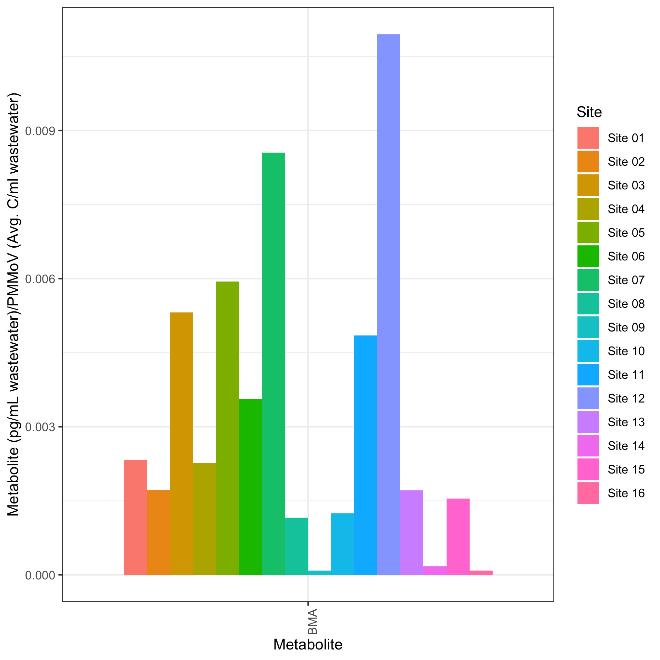

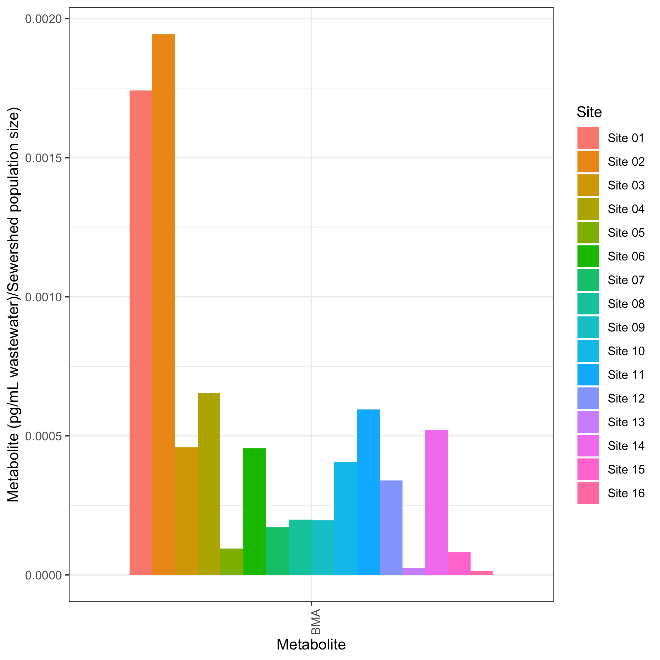


A B

# **Figure S9**. BMA concentration normalized by (A) PMMoV and (B) population size of the studied sewershed. N=64


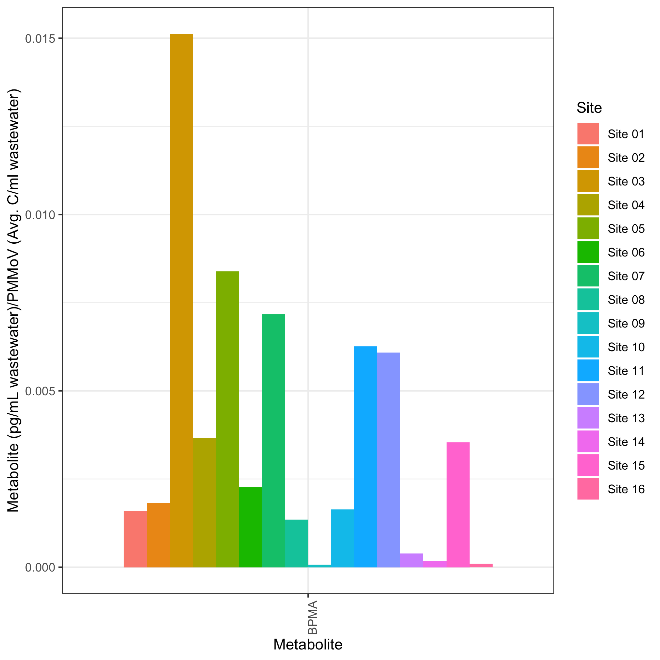

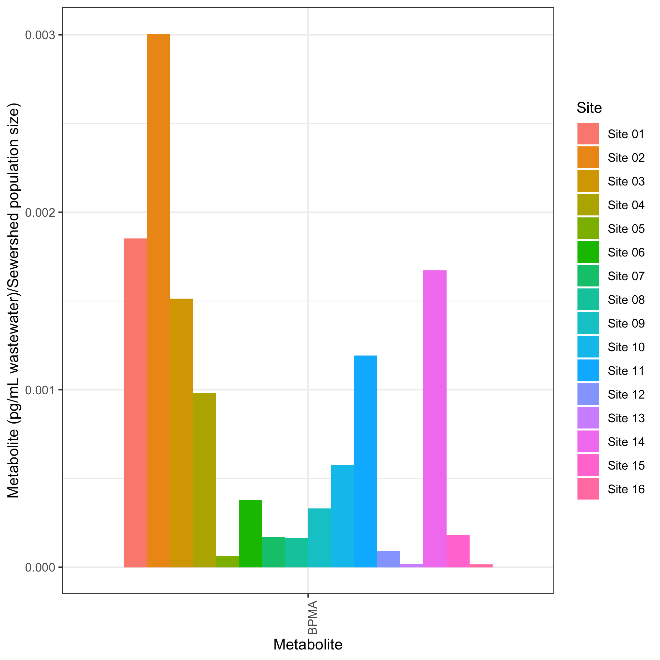


A B

# **Figure S10**. 1PMA concentration normalized by (A) PMMoV and (B) population size of the studied sewershed. N=64


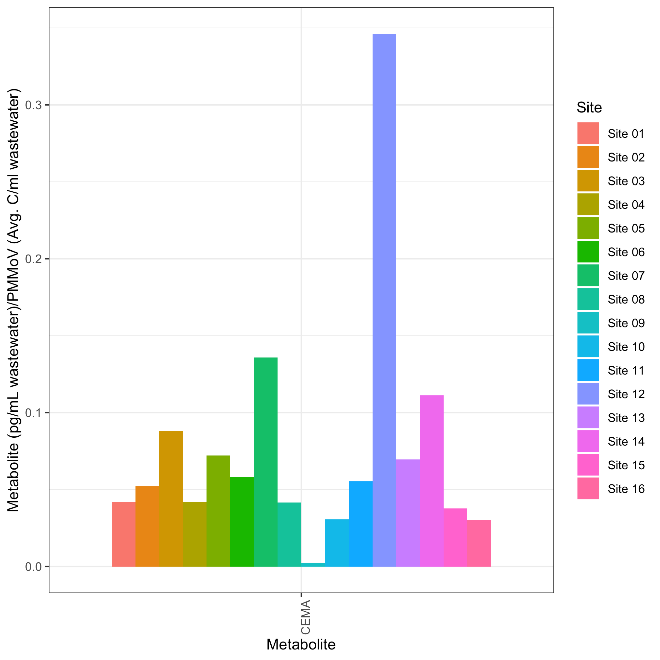

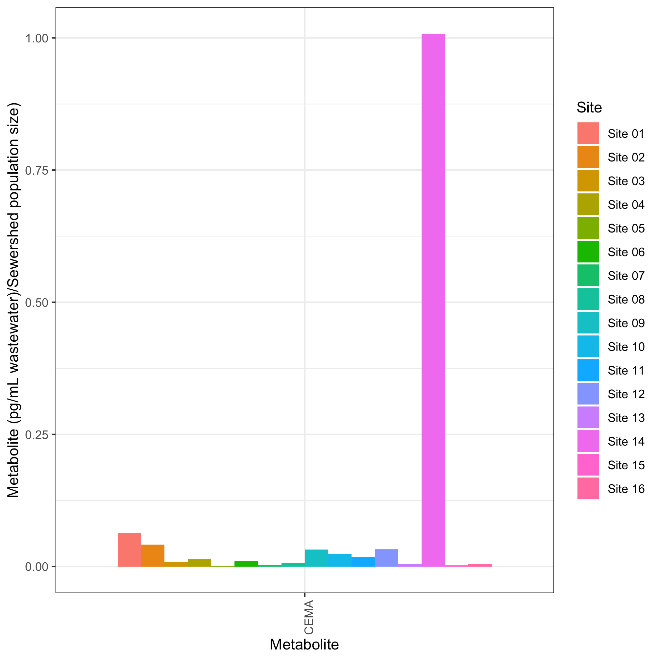


A B

# **Figure S11**. 2CoEMA concentration normalized by (A) PMMoV and (B) population size of the studied sewershed. N=64


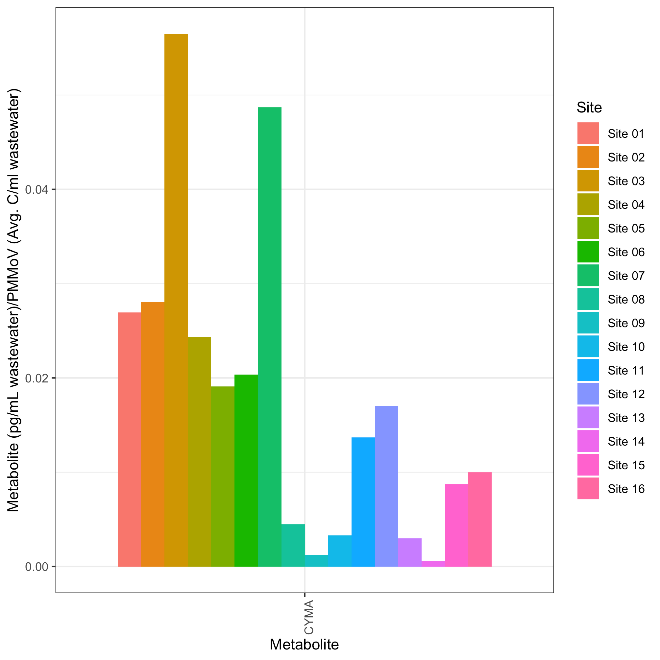

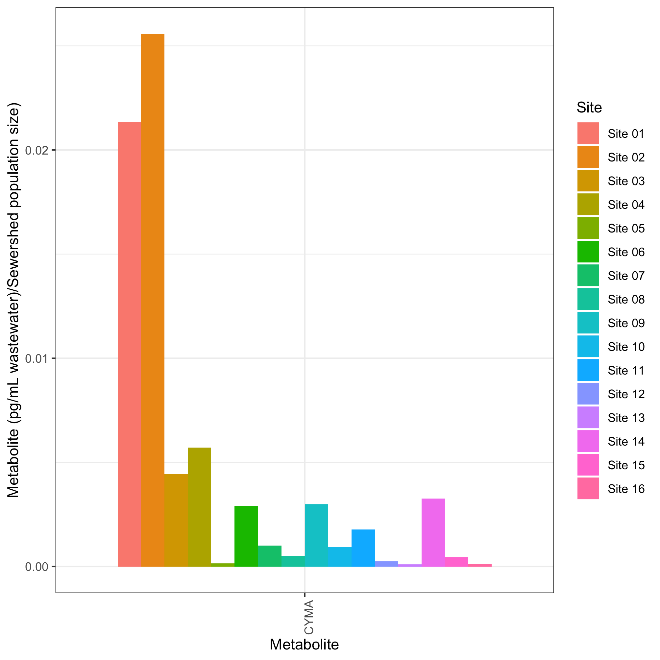


A B

# **Figure S12**. 2CyEMA concentration normalized by (A) PMMoV and (B) population size of the studied sewershed. N=64


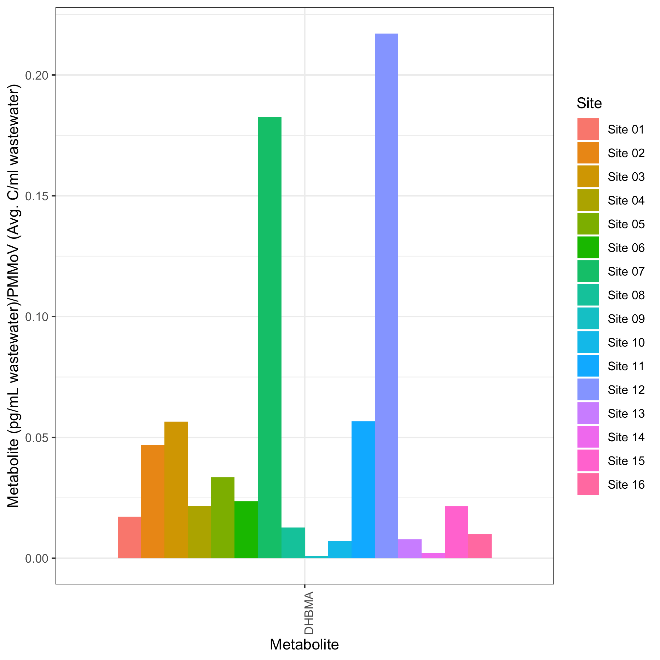

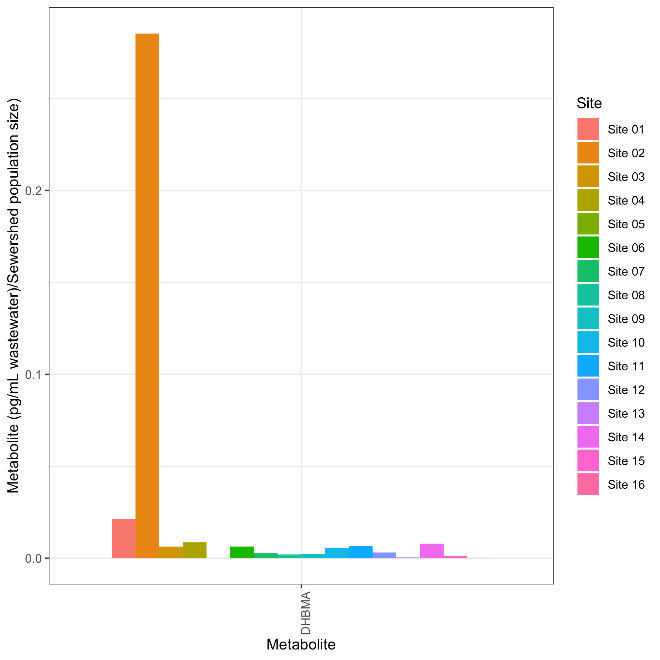


A B

# **Figure S13**. 34HBMA concentration normalized by (A) PMMoV and (B) population size of the studied sewershed. N=64


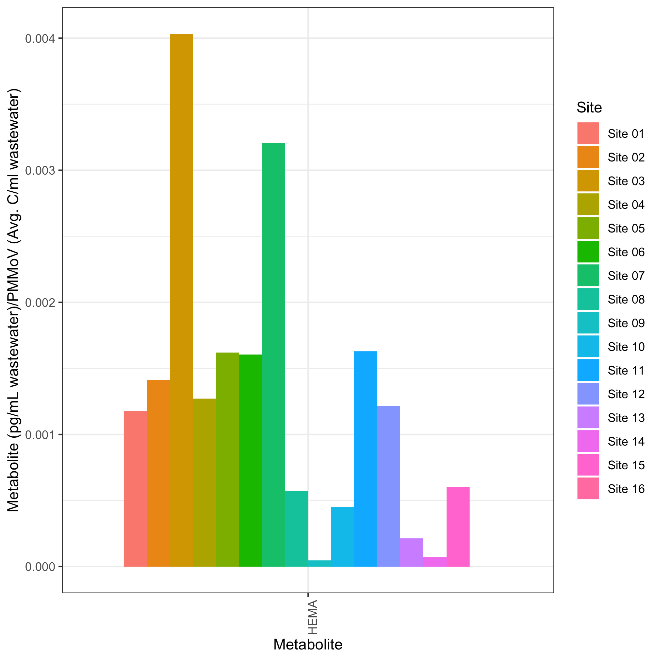

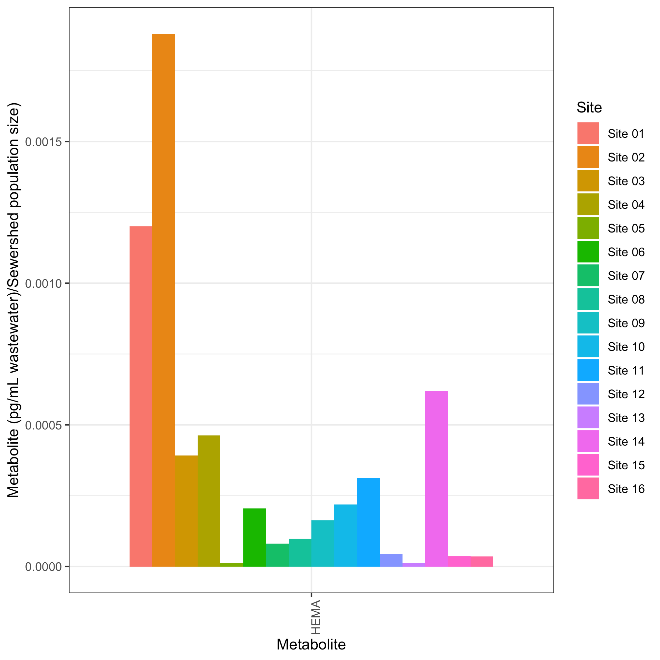


A B

# **Figure S14**. 2HEMA concentration normalized by (A) PMMoV and (B) population size of the studied sewershed. N=64


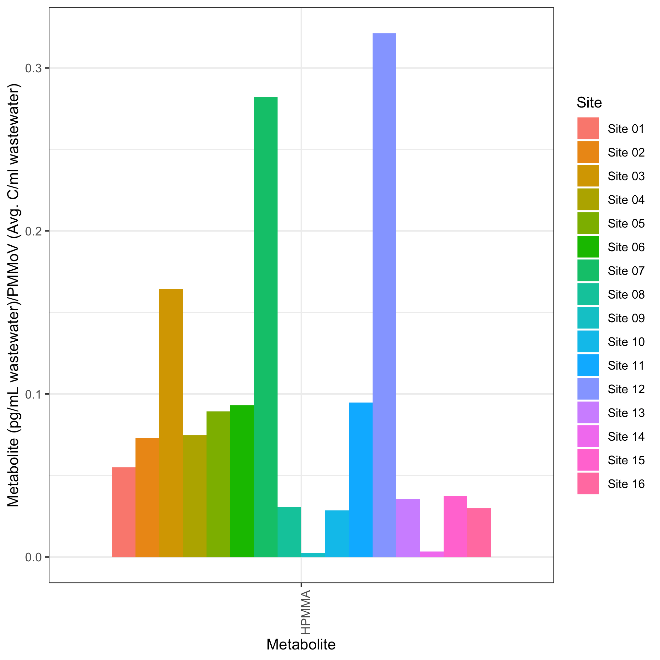

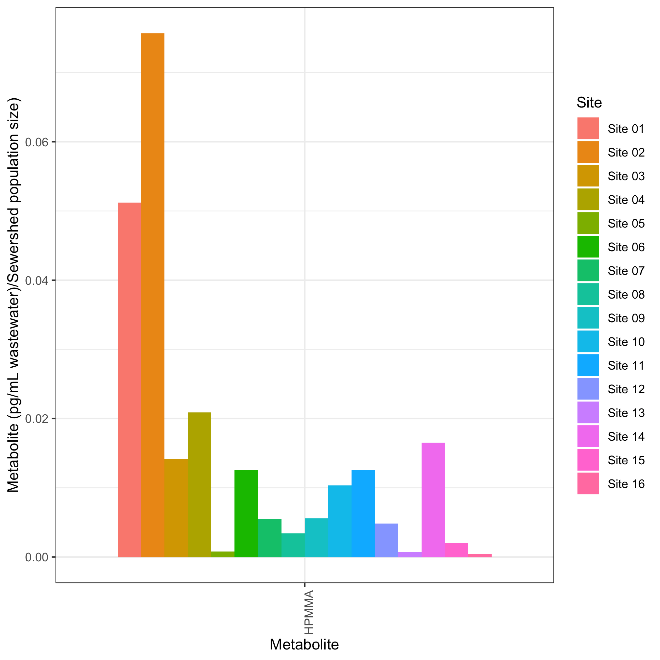


A B

# **Figure S15**. HMPMA concentration normalized by (A) PMMoV and (B) population size of the studied sewershed. N=64


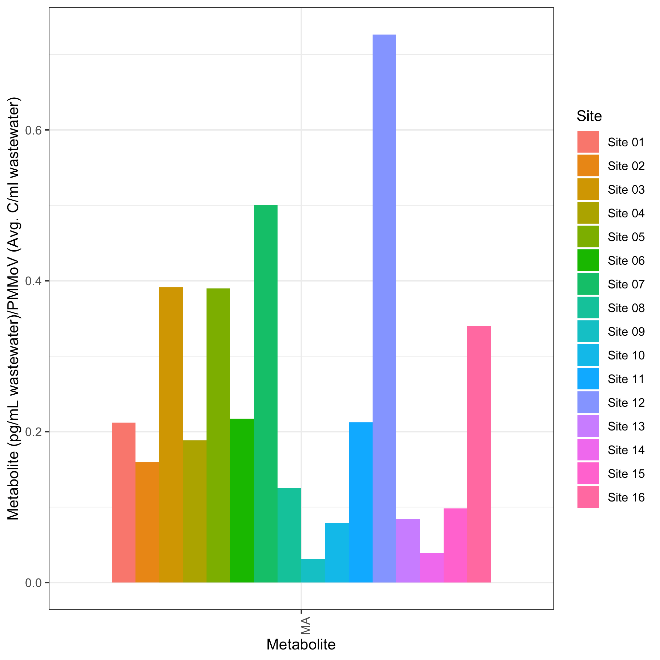

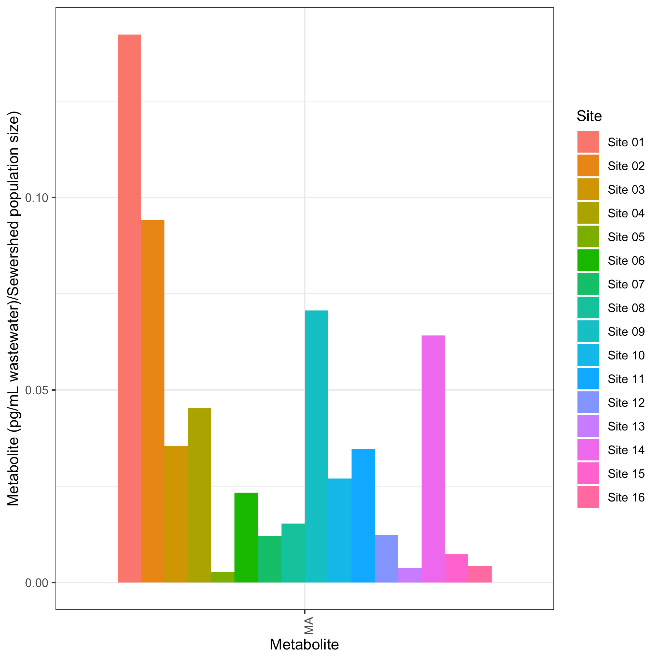


A B

# **Figure S16**. MADA concentration normalized by (A) PMMoV and (B) population size of the studied sewershed. N=64


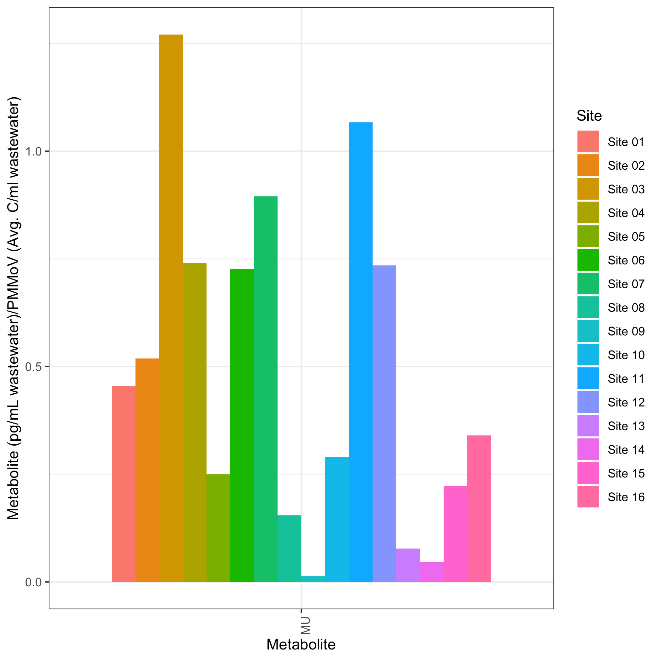

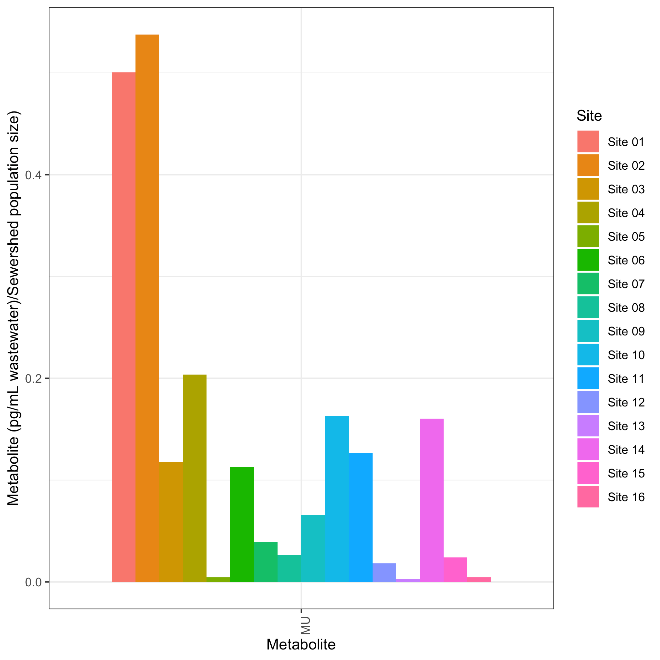


A B

# **Figure S17**. MUCA concentration normalized by (A) PMMoV and (B) population size of the studied sewershed. N=64


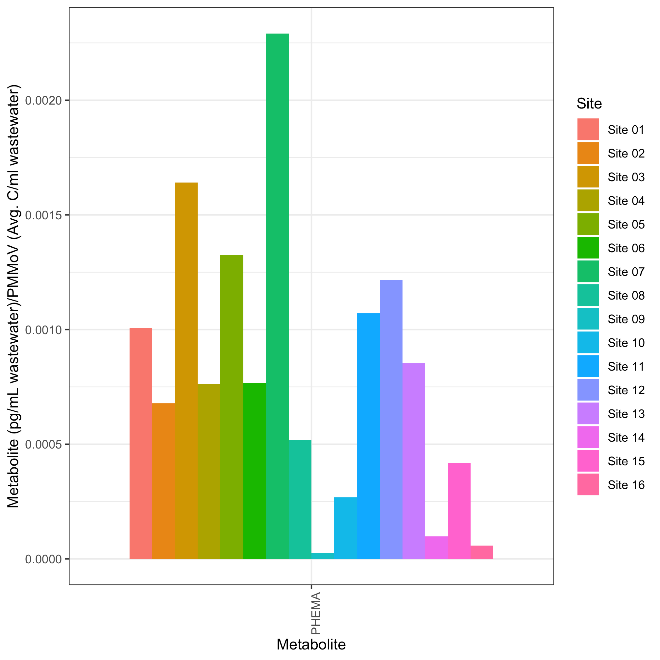

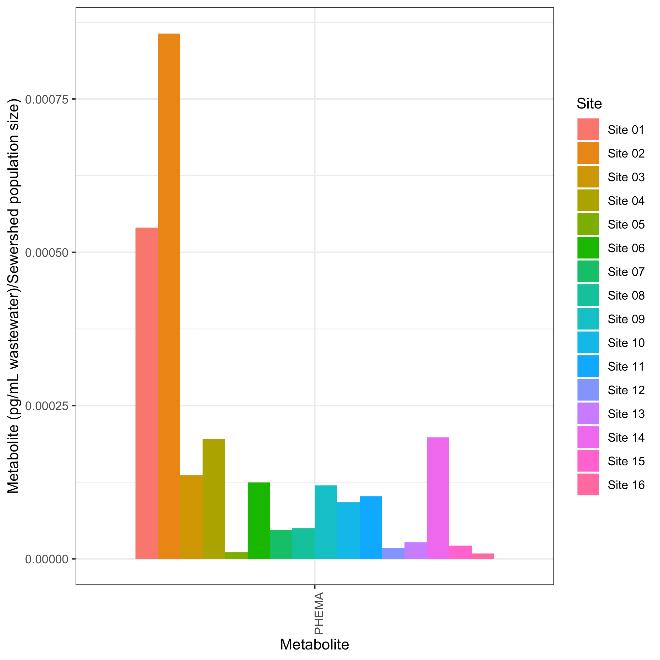


A B

# **Figure S18**. PHEMA concentration normalized by (A) PMMoV and (B) population size of the studied sewershed. N=64
